# RNA-DNA interactomes of three prokaryotes uncovered by proximity ligation

**DOI:** 10.1038/s42003-023-04853-8

**Published:** 2023-04-29

**Authors:** Alexey A. Gavrilov, Grigory S. Evko, Aleksandra A. Galitsyna, Sergey V. Ulianov, Tatiana V. Kochetkova, Alexander Y. Merkel, Alexander V. Tyakht, Sergey V. Razin

**Affiliations:** 1 Institute of Gene Biology, Russian Academy of Sciences, 119334 Moscow, Russia; 2 Massachusetts Institute of Technology, Cambridge, MA 02139 USA; 3 Faculty of Biology, Lomonosov Moscow State University, 119991 Moscow, Russia; 4 Winogradsky Institute of Microbiology, Federal Research Center of Biotechnology, Russian Academy of Sciences, 117312 Moscow, Russia

**Keywords:** Bacterial transcription, Archaeal genomics, Bacterial genomics, Next-generation sequencing, Non-coding RNAs

## Abstract

Proximity ligation approaches, which are widely used to study the spatial organization of the genome, also make it possible to reveal patterns of RNA-DNA interactions. Here, we use RedC, an RNA-DNA proximity ligation approach, to assess the distribution of major RNA types along the genomes of *E. coli*, *B. subtilis*, and thermophilic archaeon *T. adornatum*. We find that (i) messenger RNAs preferentially interact with their cognate genes and the genes located downstream in the same operon, which is consistent with polycistronic transcription; (ii) ribosomal RNAs preferentially interact with active protein-coding genes in both bacteria and archaea, indicating co-transcriptional translation; and (iii) 6S noncoding RNA, a negative regulator of bacterial transcription, is depleted from active genes in *E. coli* and *B. subtilis*. We conclude that the RedC data provide a rich resource for studying both transcription dynamics and the function of noncoding RNAs in microbial organisms.

## Abstract (web summary)

Mapping of RNA-DNA interactions in two bacteria and one archaeon using RedC, an RNA-DNA proximity ligation approach, uncovers major features that broadly characterize prokaryotic gene expression.

## Introduction

The last two decades have been marked by the development of DNA-DNA proximity ligation approaches, known as C-methods, which enabled deciphering the spatial organization of the genome across all domains of life, from bacteria to humans^1–3^. In genomes of higher eukaryotes, various types of contact domains (topologically associating domains (TADs) and chromatin compartments) have been identified^4,5^, and numerous spatial contacts between promoters and remote regulatory elements have been documented^6,7^. Contact domains have also been found in prokaryotic genomes, although their functional significance is currently unclear^8^. The application of C-methods in metagenomics has improved metagenomic assembly and allowed the study of plasmid-microbe and phage-microbe associations^9–11^.

More recently, RNA–DNA proximity ligation-based methods have entered the scene, being proved useful in the analysis of the genomic distribution of various noncoding RNAs, such as XIST, MALAT, NEAT, RoX, enhancer RNA, and spliceosomal RNAs, in several eukaryotic species, including mammals, flies, and plants^12–17^. Using RedC, an RNA–DNA proximity ligation technique developed in our laboratory, we identified noncoding RNAs associated with active and repressed chromatin in human cells and revealed the kinetics of co-transcriptional splicing^16^. We also combined RedC with the chromatin immunoprecipitation assay and identified noncoding RNAs involved in Polycomb targeting and establishing CTCF-dependent chromatin loops^18^.

Here, we used RedC to analyze the RNA–DNA interactomes of two bacteria and one archaeon. We studied the distribution of noncoding RNAs along the genome and demonstrated that in both bacteria and archaea, ribosomal RNAs are enriched at active protein-coding genes, indicating the coupling between transcription and translation. We have also shown that in bacteria, 6S noncoding RNAs are depleted from active protein-coding genes, which is consistent with their negative role in the regulation of bacterial transcription. Finally, we use RNA–DNA interaction data to trace the movement of polycistronic messenger RNAs along operons during transcription.

## Results and discussion

### Applying RedC to microbial cells

**Fig. 1:**
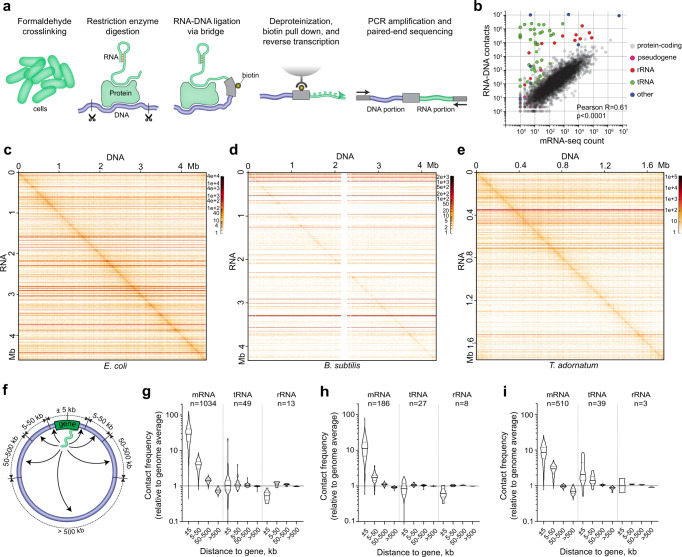
RNA–DNA interactomes of *E. coli*, *B. subtilis*, and *T. adornatum* identified by RedC. **a** Schematic of RedC protocol. **b** Correlation between the number of RNA–DNA contacts and mRNA-seq signal for *E. coli*. Pearson correlation coefficient was calculated for mRNAs. **c**–**e** Whole-genome RNA–DNA contact maps for *E. coli* (**c**), *B. subtilis* (**d**), and *T. adornatum* (**e**). **f** Scheme showing analyzed genomic intervals. **g**–**i** Frequency of contacts of mRNAs, tRNAs, and rRNAs in indicated genomic intervals relative to the average contact frequency of respective RNAs in the genome for *E. coli* (**g**), *B. subtilis* (**h**), and *T. adornatum* (**i**).

The RedC experimental protocol for mapping RNA–DNA interactions is based on formaldehyde fixation of RNA–protein–DNA complexes in vivo followed by ligation of RNA and DNA fragments present in one nucleoprotein complex via a biotinylated bridge adapter with subsequent identification of ligated fragments using paired-end sequencing^16^ (Fig. 1a and Supplementary Fig. 1). RedC reports the sites of chromosomal location simultaneously for all RNA molecules present in the cell. We applied RedC to classical laboratory bacteria *Escherichia coli* and *Bacillus subtilis* in a stationary growth phase, as well as to the thermophilic archaeon *Thermofilum adornatum*^19^ in an exponential growth phase, to test the applicability of the protocol for mapping RNA–DNA interactions under various experimental conditions. For two biological replicates of experiments with *E. coli, B. subtilis*, and *T. adornatum*, 656 M, 230 M, and 118 M paired-end reads were obtained, respectively, yielding 67 M, 6 M, and 54 M unique RNA–DNA contacts (Supplementary Table 1). The higher yield of unique contacts for *T*. *adornatum* is due to the lower proportion of multiple mapped reads (87, 96, and 18% for *E*. *coli*, *B*. *subtilis*, and *T*. *adornatum*, respectively), which reflects the presence of only one copy of 23S, 5S, and 16S rRNA genes in the *T*. *adornatum* reference genome. This increases the yield of uniquely mapped rRNA fragments. In contrast, the presence of seven and ten rRNA operons with a similar sequence in the genomes of *E*. *coli* and *B*. *subtilis*, respectively, sharply reduces the possibility of unambiguous mapping of rRNA fragments. As a result, rRNA accounts for 9, 8, and 82% of unique RNA–DNA contacts identified for *E*. *coli*, *B*. *subtilis*, and *T*. *adornatum*, respectively (Supplementary Table 2 and Supplementary Fig. 2). Apart from rRNA, the total RNA–DNA interactome is enriched in other noncoding RNAs such as tRNA, 6S RNA, SRP RNA, and tmRNA, as well as in protein-coding transcripts (Supplementary Table 2). Notably, the number of RNA–DNA contacts for a particular mRNA is proportional to the cellular level of the transcript, as follows from the comparison of RedC data for *E*. *coli* with a published mRNA-seq dataset for the same *E*. *coli* strain at the same growth stage^20^ (Fig. 1b). Previous studies on RNA–DNA interactions in eukaryotes have also reported a correlation between the level of transcript and the number of contacts it makes in the genome^13–16^.

The number of contacts identified for individual RNAs correlated well between the biological replicates (Supplementary Fig. 3, Pearson *R* > 0.99). The same is true for the RNA–DNA contact maps (Supplementary Figs. 4–6, Pearson *R* > 0.96). Thus, we combined the contacts for the biological replicates and obtained the RNA–DNA contacts maps at 100 bp resolution (Fig. 1c–e).

### Distribution of RNAs along the genome

The contact maps are characterized by the presence of diagonal and horizontal lines. We noticed that the diagonal signals most frequently correspond to the positions of protein-coding genes, indicating that mRNAs are predominantly captured near the place of their synthesis on a chromosome, while the horizontal lines typically correspond to tRNAs and rRNAs, indicating their widespread chromosomal distribution (Supplementary Figs. 4–6). mRNAs expressed at high levels may also form horizontal lines, probably reflecting nonspecific contacts of mRNAs along the genome; however, these lines almost always show increased intensity near the diagonal, whereas the lines formed by tRNAs and rRNAs do not. To systematically analyze the preferences of different RNA types for short- and long-range interactions, we selected RNAs with a high number of genomic contacts (≥500) and assessed the frequency of their contacts in several consecutive genomic intervals measured from the encoding gene (±5 kb, 5–50 kb, 50–500 kb, and >500 kb from the middle of the gene, see Fig. 1f). The obtained frequencies were presented relative to the average contact frequency of the respective RNAs in the genome (Fig. 1g–i). mRNAs showed a clear preference for the interval where the encoding gene is located, while no such preferences were observed for tRNAs and rRNAs, except for tRNAs in *T. adornatum* (Fig. 1i). The slight enrichment of tRNAs at their parental loci in *T. adornatum* may be due to active tRNA transcription during exponential growth of this archaeon. The apparent depletion of rRNAs from their parental loci in *E. coli* and *B. subtilis* (Fig. 1g, h) is due to a poor mappability of DNA reads along the multi-copy rRNA operons, which leads to the appearance of vertical white areas in the contact map (clearly visible in Supplementary Fig. 4). When we did not take into account contacts with rRNA operons and considered contacts only with their flanking regions, the frequency of rRNA contacts in the parental interval became similar to the frequency of rRNA contacts in more remote genomic intervals (Supplementary Fig. 7a, b).

### Localization of mRNAs near encoding genes

**Fig. 2:**
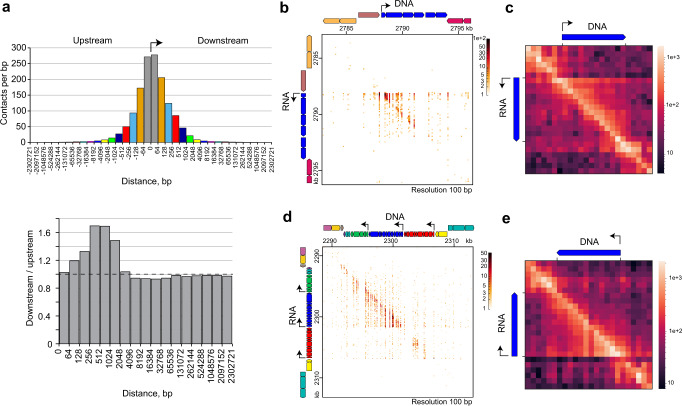
Contacts of mRNA with encoding genes and neighboring areas in *E. coli*. **a** Frequency of contacts of mRNA fragments in intervals upstream and downstream of the encoding DNA segment. Pairs of bars of the same color represent equally spaced intervals downstream and upstream of the encoding DNA segment. Shown below is the ratio of contact frequencies between equally spaced intervals. **b** RNA–DNA contact map for a region of *E. coli* genome harboring operon *rbsDACBKR* transcribed in the sense direction (colored in blue). **c** Cumulative contact map for all operons of protein-coding genes of *E. coli* transcribed in the sense direction (*n* = 267). **d** RNA–DNA contact map for a region of *E. coli* genome harboring ribosomal protein operons *rpsMKD-rpoA-rplQ* (green), *spc* (blue), and *S10* (red) transcribed in the antisense direction. **e** Cumulative contact map for all operons of protein-coding genes of *E. coli* transcribed in the antisense direction (*n* = 311). Note that a vertical streaked pattern in contact maps in (**b**) and (**d**) is due to the absence of NlaIII sites in many genomic bins at the used map resolution (100 bp).

We then analyzed the distribution of mRNA contacts near the encoding gene, focusing on the data from *E. coli*. First, we assessed the frequency of contacts of mRNA fragments with genomic intervals upstream and downstream of the site from which the RNA fragment was synthesized (Fig. 2a). Occasional fragmentation of RNA before bridge adapter ligation makes it possible to analyze the distribution of nascent transcripts along the transcribed area when transcription is not yet finished and the 3′ end of the RNA is in the active site of RNA polymerase. As expected, the highest interaction frequency was observed for the DNA region coding for the corresponding RNA fragment, which likely reflects the association of RNA and DNA via the transcriptional complex. The interaction frequency decreases with the increase in distance from a coding site (Fig. 2a, upper panel). Notably, at short distances, the frequency of contacts with the downstream intervals is higher than the frequency of contacts with equally spaced upstream intervals (Fig. 2a, lower panel), which can be explained by the movement of the nascent transcript with RNA polymerase along the transcribed area. The difference between downstream and upstream contact frequencies fades away at a distance >2 kb, which surpasses a median gene length in *E. coli* (0.8 kb) and approaches a median operon length (2.7 kb). This result appears to reflect the polycistronic nature of bacterial mRNAs. As a polycistronic transcript is held by a moving RNA polymerase until the end of an operon, the coding regions of this transcript will establish contacts not only with the corresponding genes but also with the genes located downstream in the same operon. The inspection of the RNA–DNA contact map confirms this model. The characteristic triangular structures are visible above the diagonal for operons transcribed in the sense direction (Fig. 2b, c) and below the diagonal for those that are transcribed in the antisense direction (Fig. 2d, e and see also Supplementary Fig. 4). Similar results were obtained for *B. subtilis* and *T. adornatum* (Supplementary Figs. 5, 6, 8).

### Contact profiles of noncoding RNAs

In contrast to mRNAs, rRNAs and tRNAs demonstrate widespread distribution and are not markedly enriched at the place of their synthesis (Fig. 1g–i and Supplementary Fig. 7). The absence of pronounced enrichment at the parental gene may reflect the high stability of tRNA and rRNA molecules and their rapid release from the parental gene upon transcription termination and diffusion to the place of functioning. For example, rRNA might be rapidly assembled into ribosomal particles and relocate toward active genes to take part in the co-transcriptional translation of mRNAs. To test this hypothesis, we determined the number of contacts rRNA established with the bodies of protein-coding genes and compared it with the transcriptional activity of the genes. For correct analysis, we had to take into consideration that the number of contacts determined for different DNA regions may vary depending on technical factors such as region-specific efficiency of cross-linking and restriction enzyme digestion, as well as DNA read mappability at a given genomic location. Moreover, longer genes were expected to produce more ligation products with any RNA than shorter ones. To account for differences in the gene length and the efficiency of the RedC procedure for different DNA regions, the number of rRNA contacts with a gene was normalized by the number of nonspecific contacts established with this gene by mRNA molecules produced from remote genomic regions (>250 kb from the gene). The idea to use contacts of mRNAs that “came from afar” for estimating nonspecific background ligation has been leveraged previously in the analysis of RNA–DNA interactomes of eukaryotic organisms^13,16^. Finally, as a measure of the transcriptional activity of a protein-coding gene, we calculated the number of contacts that mRNA transcribed from this gene establishes with the gene and its flanking regions (±5 kb of the middle of the gene). We reasoned that mRNA fragments captured near the gene may better convey the transcriptional activity of the gene than, for example, the total RNA output as determined by RNA-seq.

**Fig. 3:**
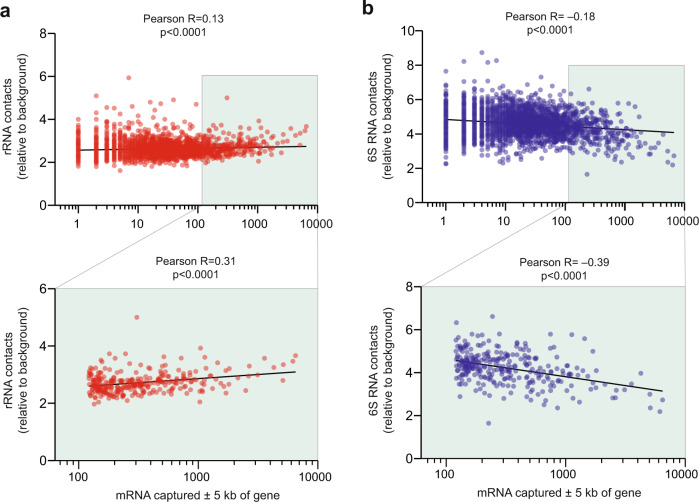
Contacts of rRNA and 6S RNA with protein-coding genes in *E. coli*. **a** Background-normalized contacts of rRNA with individual protein-coding genes (Y-axis) vs the number of contacts of respective mRNAs within ±5 kb from the middle of the gene (X-axis). The scatter plot below presents the results for the rightmost 10% points of the scatter plot above. The black lines—linear regression. **b** The same as (**a**) for 6S RNA.

In agreement with our hypothesis, we found a positive correlation between the number of rRNA contacts and the transcriptional activity of protein-coding genes. The correlation is more evident for the top 10% of most active genes in *E. coli* (Fig. 3a, lower panel), although for all protein-coding genes, a significant correlation is also observed (Fig. 3a, upper panel). Assuming that the association of rRNA with protein-coding genes reflects the presence of ribosomes on nascent transcripts, one may conclude that the transcripts of the most active protein-coding genes are translated with approximately the same efficiency. At the same time, large fluctuations of rRNA contact numbers observed for weakly expressed protein-coding genes could indicate the presence of stalled ribosomes. We also observed a weak, but significant, correlation between the number of rRNA contacts and gene activity in the analysis of the top 10% most active genes of *B. subtilis* (Supplementary Fig. 9a) and all protein-coding genes of *T. adornatum* (Supplementary Fig. 10), which argues for the coupling between transcription and translation not only in bacteria^21^ but also in archaea^22^. As a direction for future research, it would be interesting to explore if the contacts of rRNA with protein-coding genes are also correlated with the translational rate of the corresponding mRNAs. The relatively weak correlations observed in our data may reflect the fact that certain mRNAs migrate for localized translation to specific cellular locations (such as the membrane or poles) where the proteins encoded by these mRNAs are required, rather than staying close to transcription sites^23–25^. Notably, in eukaryotes, where transcription and translation are physically separated by the nuclear membrane, rRNA preferentially interacts with inactive genes, likely reflecting the synthesis of rRNA in the nucleolus around which the repressed chromatin is accumulated^16,26^.

In contrast to rRNA, 6S noncoding RNA, which is involved in the repression of σ70-dependent transcription of bacterial genes in the stationary phase^27,28^, was found to be depleted from active protein-coding genes in *E. coli* (Fig. 3b) and B*. subtilis* (Supplementary Fig. 9b).

## Conclusions

In sum, the results of the present study demonstrate the validity of the RedC protocol for studying RNA–DNA interactomes of microbial species. Using this protocol, we observed the presence of polycistronic transcriptional units and provided new evidence for co-transcriptional translation in both bacteria and archaea. The application of RNA–DNA proximity ligation techniques to bacteria and archaea provides a new high-throughput means to study transcription mechanisms and the functions of noncoding RNAs in these diverse microorganisms. These techniques can also be used in microbiome studies to identify regulatory RNAs within individual microbial species on a community-wide level, explore possible DNA/RNA-mediated associations between species, assess the metatranscriptome, and detect events of infection of bacteria by RNA-containing bacteriophages.

## Methods

### Cell strains

*E. coli* DH5α and *B*. *subtilis* 168 strains were grown overnight in Luria-Bertani broth at 37 °C and 180 rpm to a final OD600 ~1.7. *B*. *subtilis* strain 168 was kindly provided by Dr. S.A. Dubiley (Institute of Gene Biology, Russian Academy of Sciences, Moscow, Russia). *T. adornatum* strain 1910b^T^ was grown on strictly anaerobic modified Pfennig medium supplemented with 0.1 g/L of yeast extract, 1/100 (v/v) of sterile culture broth filtrate of *Desulfurococcus* sp. 1910a, and 1.0 g/L of glucose as the substrate under N_2_ in the gas phase at 80 °C and pH 5.75 to a final growth yield of 3 × 10^7^ cells/mL.

### RedC procedure

RedC procedure was performed as described previously in ref. ^16^ with minor modifications. We used 2.5 mL of an overnight culture of *E. coli* and *B*. *subtilis* and 300 mL of *T. adornatum* culture per experiment. Cells were centrifuged for 10 min at 3200 × *g* and room temperature for *E. coli* and *B*. *subtilis* and for 15 min at 7200 × *g* and room temperature for *T. adornatum*. Cells were washed with PBS, centrifuged again, and fixed in 3% formaldehyde (Sigma-Aldrich F8775) in PBS for 30 min at room temperature, followed by quenching with 375 mM glycine. Cells were centrifuged at 4 °C as above, washed with cold PBS, and centrifuged again.

Cells were resuspended in 1 mL PBS supplemented with 1× protease inhibitors (Bimake) and 200 U SUPERase.In RNase inhibitor (Invitrogen) and mechanically disrupted using a bead beater homogenizer (MP Biomedicals FastPrep-24) in 2 mL Lysing Matrix A tube at 6.0 m/s for 25 s at room temperature. Cells were centrifuged for 10 min at 16,100 × *g* and 4 °C and resuspended in 500 µL nuclease-free water (Qiagen) supplemented with 1× protease inhibitors (Bimake) and 100 U SUPERase.In RNase inhibitor (Invitrogen) followed by adding 15 µL 10% SDS and incubating for 30 min at 37 °C with shaking at 1200 rpm. SDS was sequestered by adding 50 µL 20% Triton X-100 followed by incubation for 30 min at 37 °C with shaking at 1200 rpm. After adding 200 µL warm 4× NEB buffer 4, the insoluble fraction containing the crosslinked chromatin was pelleted for 10 min at 16,100 × *g* at room temperature and resuspended in 500 µL 1× NEB buffer 4. DNA was digested with 20 µL NlaIII (10 U/µL, NEB) for 3 h at 37 °C with shaking at 1200 rpm.

Digested chromatin was pelleted for 10 min at 16,100 × *g* and 10 °C and resuspended in 150 µL 1× NEB buffer 2, followed by mixing with 300 µL AMPure XP magnetic beads (Beckman Coulter). Beads with immobilized chromatin were collected on a magnet, washed twice with 1 mL 80% ethanol, and air-dried for 1 min. 3′ P ends of RNA were dephosphorylated by resuspending bead-chromatin complexes in 142.5 µL dephosphorylation solution (1× PNK buffer (NEB), 0.1% Triton X-100, hereinafter the concentration is given as for the enzyme-containing mixture) followed by adding 7.5 µL PNK (10 U/µL, NEB). The mixture was incubated for 30 min at 37 °C with shaking at 900 rpm. Bead-chromatin complexes were pelleted for 10 min at 16,100 × *g* and 10 °C and resuspended in 189 µL blunting solution (1× T4 DNA ligase buffer (NEB), 0.25 mM each dNTP). The mixture was supplemented with 5 µL DNA polymerase (3 U/µL, NEB) and 6 µL Klenow (5 U/µL, NEB), and DNA blunting was carried out for 1 h at 22 °C with shaking at 900 rpm. Bead-chromatin complexes were pelleted as above, resuspended in 200 µL nuclease-free water, and mixed with 200 µL AMPure buffer (20% PEG-8000, 2.5 M NaCl). Bead-chromatin complexes were collected on a magnet, washed twice with 1 mL 80% ethanol, and air-dried for 1 min. Bead-chromatin complexes were washed with 200 µL 1× NEB buffer 2 supplemented with 1% Triton X-100, pelleted for 10 min at 16,100 × *g* and 10 °C, and resuspended in 198 µL A-tailing solution (1× NEB buffer 2, 0.5 mM dATP, 1% Triton X-100). DNA ends were A-tailed by adding 1.5 µL Klenow (exo-) (50 U/µL, NEB) followed by incubation for 1 h at 37 °C with shaking at 900 rpm. Bead-chromatin complexes were subsequently washed with 200 µL 1× NEB buffer 2 supplemented with 1% Triton X-100, with 200 µL 1× RNA ligase buffer (NEB) supplemented with 0.1% Triton X-100, and with 200 µL 1× RNA ligase buffer (NEB) by repeating resuspending/pelleting.

The 3′ OH ends of RNA were ligated with 5′ rApp ends of the bridge adapter (Supplementary Table 3). For this purpose, bead-chromatin complexes were resuspended in 142.5 µL RNA ligase solution (1× RNA ligase buffer (NEB), 4.5 µM bridge adapter, 20% PEG-8000 (NEB)), 7.5 µL T4 RNA ligase 2 truncated (200 U/µL, NEB) were added, and the mixture was incubated for 6 h at room temperature then overnight at 16 °C with shaking at 900 rpm. To wash off the non-ligated bridge adapter, bead-chromatin complexes were pelleted for 10 min at 16,100 × *g* and 10 °C, resuspended in 200 µL nuclease-free water, and mixed with 165 µL AMPure buffer. Bead-chromatin complexes were collected on a magnet, washed twice with 1 mL 80% ethanol, and air-dried for 1 min. Bead-chromatin complexes were washed with 200 µL 1× RNA ligase buffer (NEB), pelleted as above, and resuspended in 95 µL PNK solution (1× T4 DNA ligase buffer (NEB), 0.1% Triton X-100). Then, 5 µL PNK (10 U/µL, NEB) were added, and the mixture was incubated for 1 h at 37 °C with shaking at 900 rpm. Bead-chromatin complexes were pelleted as above, resuspended in 980 µL 1.02× T4 DNA ligase buffer (Thermo Scientific), followed by adding 20 µL T4 DNA ligase (5 Weiss U/µL, Thermo Scientific). DNA proximity ligation was allowed to proceed for 6 h at 22 °C with rotation followed by pelleting bead-chromatin complexes as above.

To reverse formaldehyde cross-links and digest proteins, bead-chromatin complexes were resuspended in 470 µL proteinase K solution (100 mM NaCl, 10 mM Tris pH 7.5, 2 mM EDTA, 1% SDS), 30 µL proteinase K (20 mg/mL, Ambion) were added, and incubation for 1 h at 55 °C and then for 2 h at 65 °C followed. To precipitate RNA–DNA chimeras, 3 μL GlycoBlue (Thermo Scientific), 50 μL 3 M NaAC, and 550 μL isopropanol were added, and after overnight incubation at −80 °C, the mixture was centrifuged for 30 min at 16,100 × *g* and 4 °C. The pellet was resuspended in 200 μL nuclease-free water, and RNA–DNA chimeras were further purified with 0.75 volumes of AMPure XP beads and finally eluted into 100 μL nuclease-free water, followed by measuring the concentration with a Qubit dsDNA broad range kit.

RNA–DNA chimeras (4 μg) were digested with MmeI in a 120-μL reaction containing 1× NEB buffer 4, 0.1 mg/mL BSA (NEB), 80 μM SAM (NEB), 0.1 μM ds oligo with MmeI site (Supplementary Table 3), and 5 U MmeI (NEB) for 2 h at 37 °C. The recognition site of MmeI is incorporated into the bridge adapter to uniformly generate DNA portions of 18–20 nt. A short ds oligo containing the MmeI recognition site is added to stimulate the cleavage of DNA molecules containing a single MmeI site.

After MmeI digestion, RNA–DNA chimeras were subjected to biotin pull-down. For this process, 7.5 μL of Dynabeads MyOne Streptavidin C1 beads (10 mg/mL, Thermo Scientific) were washed twice with 400 μL tween washing buffer (TWB) (5 mM Tris pH 7.5, 0.5 mM EDTA, 1 M NaCl, and 0.05% Tween 20) by repeating the resuspension/magnet separation. Streptavidin beads were resuspended in 120 μL 2× binding buffer (10 mM Tris pH 7.5, 1 mM EDTA, 2 M NaCl) and mixed with the solution after MmeI digestion followed by incubation for 15 min at room temperature to bind the biotinylated bridge to streptavidin beads. Streptavidin beads with tethered RNA–DNA chimeras were washed twice with 600 μL TWB, once with 100 μL 1× NEB buffer 2, once with 50 μL 1× First-Strand Buffer (Clontech), and resuspended in 38 μL reverse transcriptase solution (1× First-Strand Buffer (Clontech), 2.5 mM DTT (Clontech), 1 mM each dNTP, 1 μM switch template oligo (Supplementary Table 3), and 20 U SUPERase.In RNase inhibitor (Invitrogen)). After preheating at 42 °C for 2 min, reverse transcription was initiated from the bridge 3′ OH by adding 2 μL SMARTScribe Reverse Transcriptase (100 U/μL, Clontech) followed by incubation for 1 h at 42 °C with shaking at 800 rpm. Reverse transcriptase first transcribes bridge DNA, then DNA-RNA junction, then RNA. Upon reaching the 5′ end of the RNA, reverse transcriptase adds a few non-template nucleotides (predominantly dC) to the 3′ end of cDNA. This dC stretch pairs with rGrGrG of the switch template oligo, and reverse transcriptase continues replication using the switch template oligo as a template (SMART technology). The switch template oligo provides a site for the annealing of the Illumina-indexed primer^16^.

After cDNA synthesis, streptavidin beads were washed twice with 600 μL TWB, once with 100 μL 1× NEB buffer 2, once with 100 μL 1× T4 DNA ligase buffer (Thermo Scientific), and resuspended in 48 μL DNA ligase solution (1× rapid ligation buffer (Thermo Scientific), 3 μM NN-adapter (Supplementary Table 3). Then, 2 µL T4 DNA ligase (5 Weiss U/µL, Thermo Scientific) were added to ligate DNA NN ends produced by MmeI digestion to adapter NN ends, and incubation for 30 min at 22 °C followed. The NN-adapter provides a site for the annealing of the Illumina universal primer^16^. After ligation, streptavidin beads were washed twice with 600 μL TWB, once with 100 μL 1× NEB buffer 2, once with 100 μL 10 mM Tris pH 8.0, and resuspended in 12 μL water.

DNA-cDNA chimeras were amplified in 50 μL PCR containing 1× KAPA HiFi Fidelity Buffer, 0.3 mM each dNTP, 0.5 μM universal primer, 0.5 μM indexed primer (Supplementary Table 3), 1 U KAPA HiFi DNA Polymerase, and 4 μL streptavidin beads from the above step. PCR was performed as follows: 95 °C 5 min, [98 °C 20 s, 65 °C 15 s, and 72 °C 20 s] × 12 cycles for *E. coli* and *B*. *subtilis* or 15 cycles for *T. adornatum*, 72 °C 3 min. Supplementary Figure 1 presents the results of a titration PCR performed to select an optimal number of PCR cycles for the amplification of DNA-cDNA chimeras from *E. coli*. PCR products of two reactions were pooled and purified with 1.2 volumes of AMPure XP beads for *E. coli* and *B*. *subtilis* or with 1 volume of AMPure XP beads for *T. adornatum*. PCR products were paired-end sequenced on the Illumina NovaSeq platform with a read length of 100 nt.

### Read filtering and mapping

The raw RedC reads were processed using the RedClib computational pipeline (https://github.com/agalitsyna/RedClib) as described previously in ref. ^16^ with minor modifications. Briefly, forward and reverse reads were subjected to the scanning of adapters, bridges, and GGG/CCC oligonucleotides. The DNA portion was extracted as the region of the forward read upstream of the bridge, the RNA 3′ portion was extracted as the region of the forward read downstream of the bridge, and the RNA 5′ portion was extracted as the region of the reverse read downstream of the first GGG. DNA portions of 18–20 nucleotides, RNA 3′ portions of ≥10 nucleotides, and RNA 5′ portions of ≥10 nucleotides were selected for the next mapping step. Before mapping, the end of the DNA portion adjoining the bridge was supplemented with CATG (the 3′ overhang produced by NlaIII digestion and then blunted) to reestablish the original sequence. “Reattaching” CATG to DNA portions increases their length to 22–24 nucleotides, which improves the yield of unique mappings.

DNA portions, RNA 3′ portions, and RNA 5’ portions were independently mapped to the CP025520, CP053102, and CP006646 reference genomes for *E. coli*, *B*. *subtilis*, or *T. adornatum*, respectively, with the hisat2 program (-﻿-﻿no-softclip --﻿﻿no-spliced-alignment). We retained only such DNA-RNA 3′-RNA 5′ triples that were all successfully and uniquely mapped to the reference genome. If one of the portions was missing, non-uniquely mapped, or unmapped, the read pair was filtered out. Finally, to avoid artifacts of intermolecular template switching of the reverse transcriptase, we discarded cases when RNA 3′ and RNA 5′ portions were mapped far apart from each other (>10 Kb) or to the same DNA strand. Supplementary Table 1 shows the number of read pairs retained after each consecutive step of the data processing pipeline described above.

### RNA annotation

We use RNA 3′ portions retrieved from the forward reads as described above. We assign RNA 3′ portions to genes by the featureCounts program using the GCA_002848225.1 assembly for *E. coli*, the GCA_013009385.1 assembly for *B. subtilis*, and the GCA_000446015.1 assembly for *T. adornatum*. We require that the RNA 3’ portion be mapped to the strand opposite to that of the gene, as expected from the RedC procedure. Finally, we combine DNA portions mated with RNA 3′ portions originating from a single gene, thus obtaining a whole-genome contact profile for each respective RNA.

### Preferences of RNAs for short- and long-range interactions

For each RNA, we calculate the number of contacts this RNA establishes in four consecutive genomic intervals: ±5 kb of the middle of the encoding gene; 5–50 kb upstream and downstream of the middle of the encoding gene; 50–500 kb upstream and downstream of the middle of the encoding gene; and >500 kb of the middle of the encoding gene (Fig. 1f and Supplementary Data 1–3). Contacts are assigned to genomic intervals based on the mapping position of the end of the DNA portion adjoining the NlaIII site. We divide the number of contacts determined for an interval by the interval length to obtain the contact frequency. The obtained contact frequencies are presented relative to the average contact frequency of a respective RNA in the genome calculated as the ratio of the number of contacts of this RNA with the genome to the genome length. For a robust analysis, only the RNAs with ≥500 contacts with the genome are considered.

### Contacts of mRNAs with upstream and downstream intervals

We select a set of RNA–DNA contacts such that the RNA portion is mapped to the proper strand of a protein-coding gene, and the DNA portion is mapped anywhere in the genome. We calculate the distance between RNA and DNA portions d1 = |rna – dna|, where rna is the mapping coordinate of the RNA 3′ end, and dna is the mapping coordinate of the end of the DNA portion adjoining the NlaIII site. Because the genome is circular, we calculate the distance d2 = |d1 – L|, where L is the genome length, and consider the lesser of d1 and d2 as the distance at which contact occurred.

We next determine if contact occurred upstream or downstream of the RNA 3′ end mapping coordinate, with respect to the direction of transcription. A contact is assigned upstream in the following cases: RNA is transcribed in the sense direction, rna – dna >0, and d1 < d2; RNA is transcribed in the antisense direction, rna – dna <0, and d1 < d2; RNA is transcribed in the sense direction, rna – dna <0, and d1 ≥ d2; RNA is transcribed in the antisense direction, rna – dna >0, and d1 ≥ d2. A contact is assigned downstream in the following cases: RNA is transcribed in the sense direction, rna – dna <0, and d1 < d2; RNA is transcribed in the antisense direction, rna – dna >0, and d1 < d2; RNA is transcribed in the sense direction, rna – dna >0, and d1 ≥ d2; RNA is transcribed in the antisense direction, rna – dna <0, and d1 ≥ d2.

We then assign the contacts to upstream or downstream intervals of increasing length: [0, 64), [64, 128), [128, 256), [256, 512), [512, 1024), etc. Finally, we calculate the contact frequency by dividing the number of contacts in the intervals by the length of the intervals.

### Contacts of rRNA and 6S RNA with protein-coding genes

We determine the number of contacts established with a protein-coding gene by all rRNA molecules (16S, 23S, and 5S) or by 6S RNA (Supplementary Data 1–3). The obtained number of contacts is normalized by the number of nonspecific background contacts established with the same protein-coding gene by mRNA fragments produced from remote genomic regions (the distance between the mapping position of the 3′ end of an mRNA fragment and the analyzed protein-coding gene is required to be >250 kb). For a robust analysis, only protein-coding genes with a number of background contacts ≥10 are considered (3519 of 4150 protein-coding genes for *E. coli*, 3453 of 4326 protein-coding genes for *B. subtilis*, and 1141 of 1375 protein-coding genes for *T. adornatum*).

### Estimation of transcription level of protein-coding genes

To estimate the transcriptional activity of a protein-coding gene, we calculate the number of contacts that mRNA transcribed from this gene establishes near the gene (we arbitrarily use the region ±5 kb of the middle of the gene). We note that the number of contacts of individual mRNAs with this region is proportional to the total number of contacts of these mRNAs with the genome (Supplementary Fig. 11a–c) and to the level of these mRNAs in RNA-seq data^20^ (Supplementary Fig. 11d).

### Cumulative contact map for operon

We select a set of RNA–DNA contacts such that the RNA portion is mapped to the proper strand of a protein-coding gene, and the DNA portion is mapped anywhere in the genome. We divide operons of protein-coding genes with their flanking regions of half-operon length into 24 bins (12 bins for operon ± 6 bins for flanks) and plot a 24 × 24 RNA–DNA contact matrix for each operon. We then sum the contact matrices obtained for operons transcribed in the sense direction or for operons transcribed in the antisense direction.

### Statistics and reproducibility

All experiments were performed in two biological replicates. Pearson correlation coefficient analysis of RNA–DNA contact matrices (Supplementary Figs. 4–6) and RNA–DNA contact numbers for individual RNAs (Supplementary Fig. 3) showed high reproducibility of RedC experiments.

### Reporting summary

Further information on research design is available in the Nature Portfolio Reporting Summary linked to this article.

## Supplementary information

Razin_Peer Review File

Supplementary Information

Description of Additional Supplementary Files

Supplementary Data 1

Supplementary Data 2

Supplementary Data 3

Supplementary Data 4

Reporting Summary

## Data Availability

Datasets with raw fastq RedC data and processed TSV files with contacts are available under GEO accession: GSE209901. The uncropped gel image is provided as Supplementary Fig. 12. The numerical source data for graphs are available in Supplementary Data 1–4. Other data are available from the corresponding author on reasonable request.

## Code availability

The code for read processing is available as RedClib on github at https://github.com/agalitsyna/RedClib/tree/redc-bridge-variants or at 10.5281/zenodo.7843523^29^.
