# Supplementary material for: RNA-DNA interactomes of three prokaryotes uncovered by proximity ligation
Source: Commun Biol. 2023 Apr 29;6:473. doi: 10.1038/s42003-023-04853-8 (PMC10148824; doi:10.1038/s42003-023-04853-8)
Supplement: Supplementary file 2 — Supplementary Information [file 42003_2023_4853_MOESM2_ESM.pdf]

Supplementary Figures

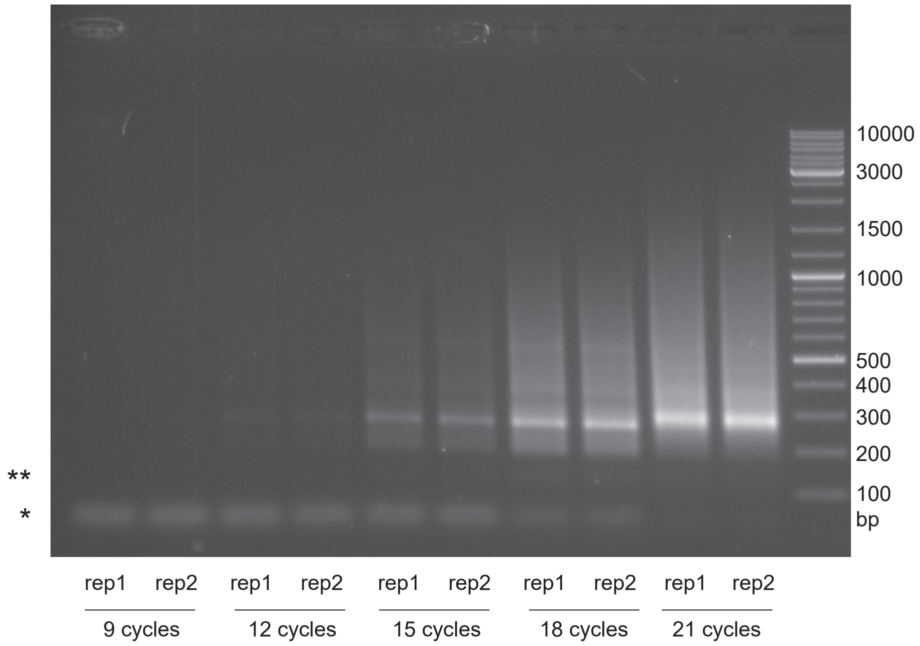

Supplementary Figure 1. Agarose gel electrophoresis of products of amplification of RNA-DNA chimeras from *E. coli* obtained after different number of PCR cycles. PCR products are expected to have a size of ≥ 181 bp (58 bp left Illumina adapter, 18-20 bp DNA portion, 37 bp bridge adapter, RNA portion of variable size, and 67 bp right Illumina adapter). Short RNA portions may represent short RNA species or originate as a result of occasional fragmentation of RNA during experimental procedure. A major band of ~ 260 bp presumably corresponds to tRNA. Asterisk shows free primer, double asterisk shows primer dimer.

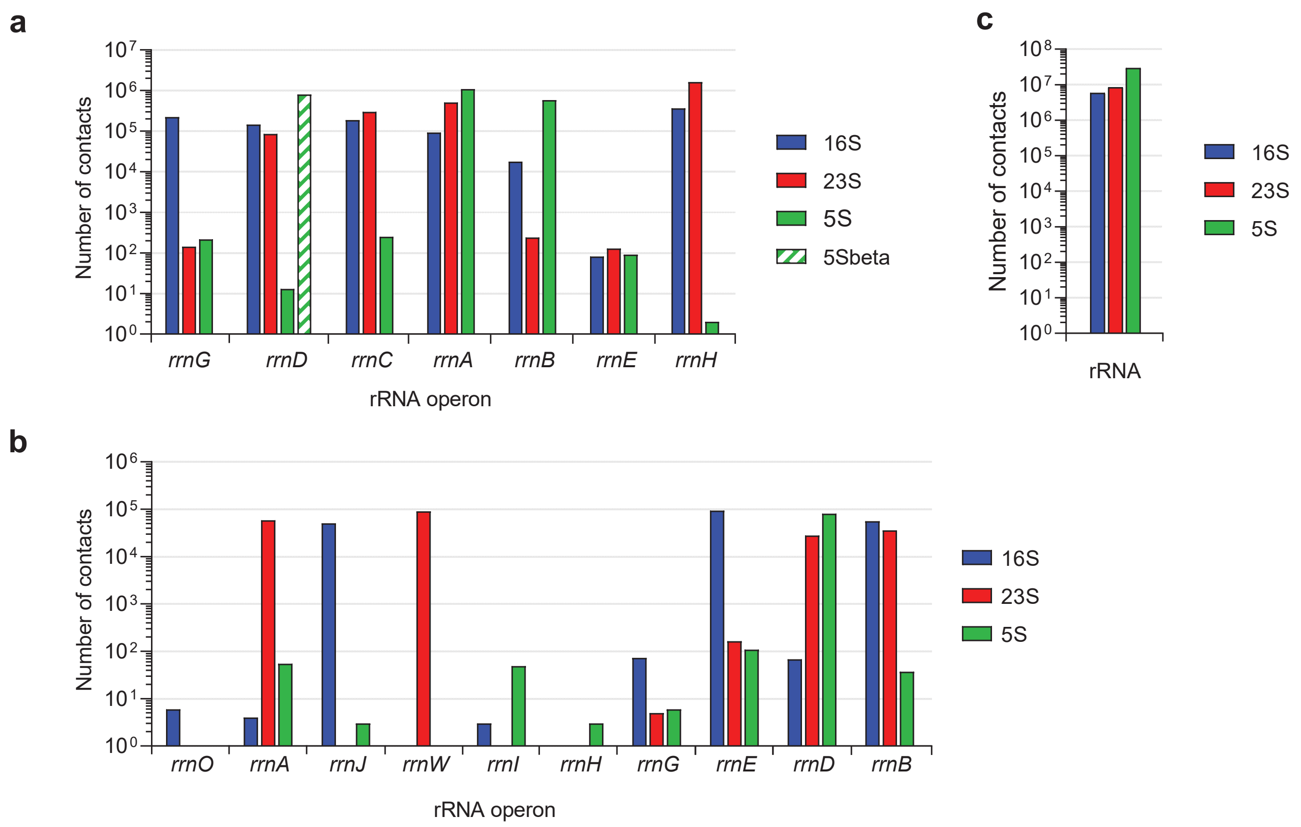

Supplementary Figure 2. Number of contacts identified for individual rRNAs in experiments with *E. coli* (a), *B. subtilis* (b) and *T. adornatum* (c). Presented is the total number of rRNA contacts in the genome. We note that our read mapping procedure filters out RNA-DNA ligation products whose RNA portion, or DNA portion, or both, map more than one time on the genome and retains only uniquely mapped RNA-DNA contacts. With this mapping procedure, a large fraction of rRNA contacts remain unidentified in *E. coli* and *B. subtilis*, because these species contain several copies of rRNA operons in their genomes (7 and 10, respectively), and many rRNA fragments present in RNA-DNA ligation products cannot be unambiguously assigned to a particular rRNA. Moreover, the used mapping procedure filters out not only many rRNA-DNA ligation products representing contacts of rRNA with different genomic regions, but also many RNA-rDNA ligation products representing contacts of different RNAs (including rRNA itself) with rRNA operons. An alternative strategy would be to allow multiple mapping and then to assign 1/7 or 1/10 of the identified rRNA contacts to each rRNA operon. However, we did not use this strategy because we wished to preserve information about the origin of rRNA fragments. The different yield of unique mappings appears to be a primary factors behind a drastic variation in the number of RNA-DNA contacts identified for different rRNAs (**a**,**b**) and for different portions of the same rRNA molecule in experiments with *E. coli* and *B. subtilis*. Note, for example, that for the *rrnB* rRNA operon of E.coli the contacts are only detected for the end of 16S rRNA (see Supplementary Fig. 4). The different expression level of rRNA operons may also contribute to the observed variation in the contact number determined for different rRNAs.

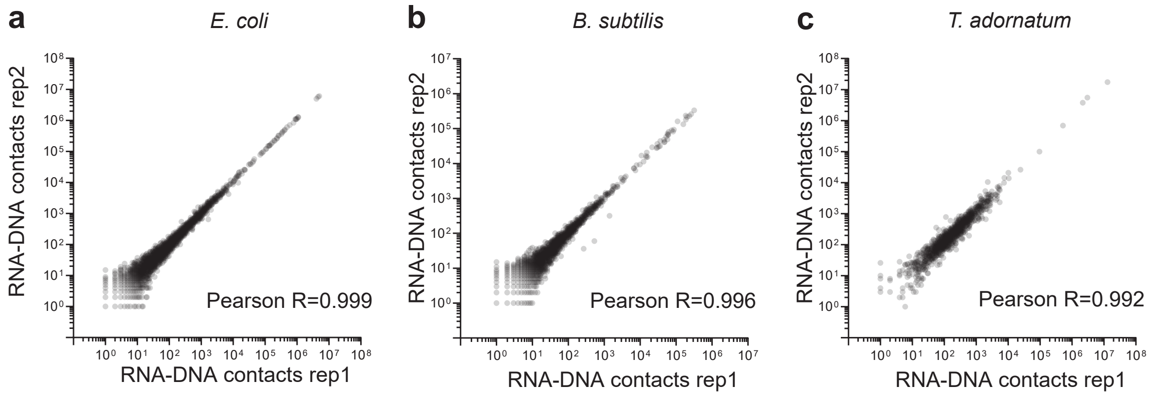

Supplementary Figure 3. Correlation of contact numbers of individual RNAs between biological replicates. Scatter plots show the total number of RNA-DNA contacts identified for each RNA in replicates of experiments with *E. coli* (**a**), *B. subtilis* (**b**) and *T. adornatum* (**c**).

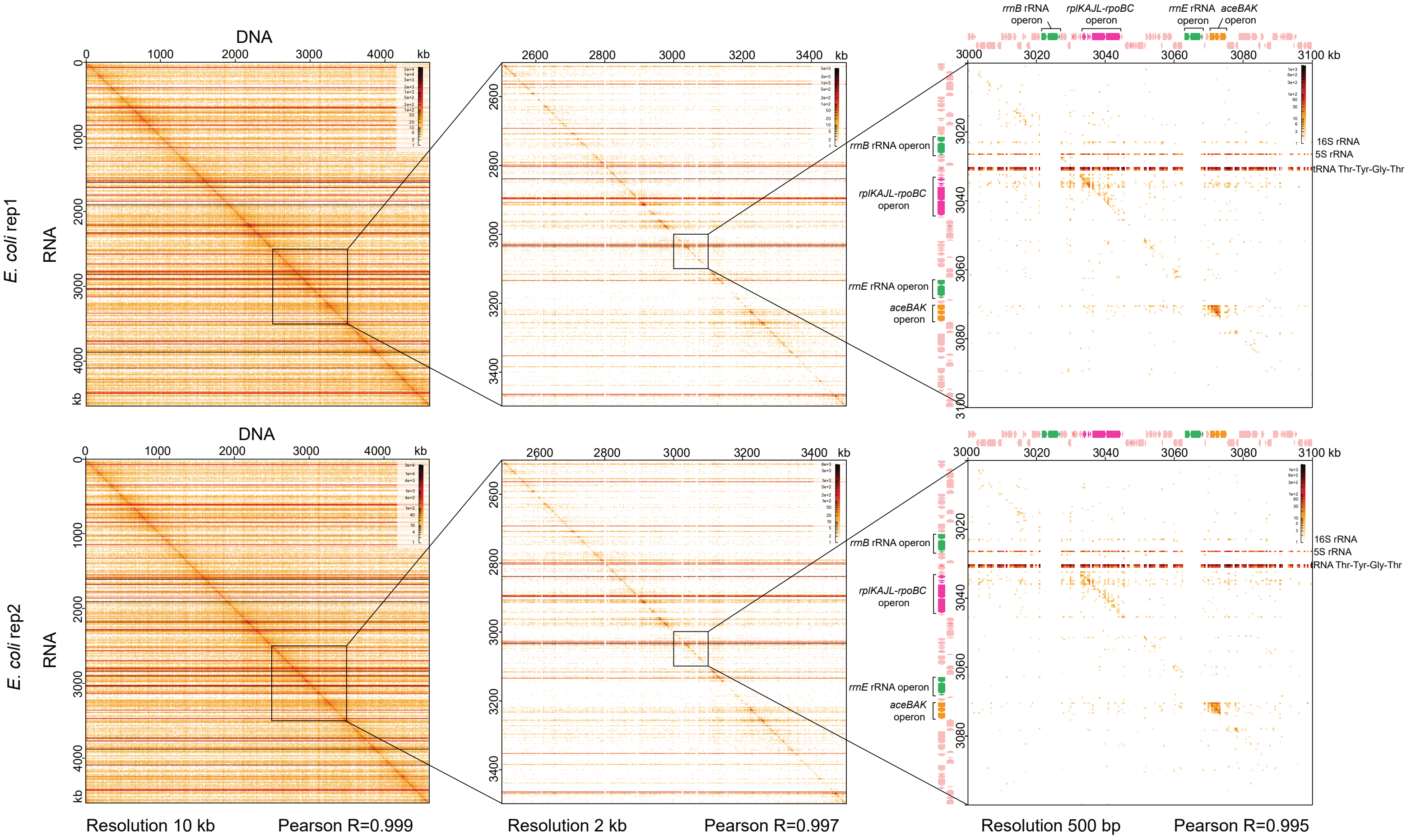

Supplementary Figure 4. RNA-DNA contact maps for biological replicates of the experiment with *E. coli.* Contact maps are demonstrated at the following scales: the whole genome (left), a 1 Mb region (middle), and a 100 kb region (right). Presented below the maps are Pearson correlation coefficients calculated for whole-genome contact maps at indicated resolution. Along the right maps, the positions of genes and operons are indicated. Note characteristic contact patterns on the right maps: triangles near diagonal at the *aceBAK* operon and the *rplKAJL-rpoBC* operon; horizontal lines at the Thr-Tyr-Gly-Thr tRNA gene cluster and the 16S and 5S rRNA genes of the *rrnB* rRNA operon. Note white horizontal areas occupying the major portion of the *rrnB* rRNA operon and the whole *rrnE* rRNA operon, indicating the absence of uniqely mapped RNA reads in these genomic regions. The ceased rRNA transcription during stationary phase may also contribute to horizontal white areas at rRNA operons. Also note white vertical areas at the both rRNA operons, indicating the absence of uniquely mapped DNA reads in these genomic regions.

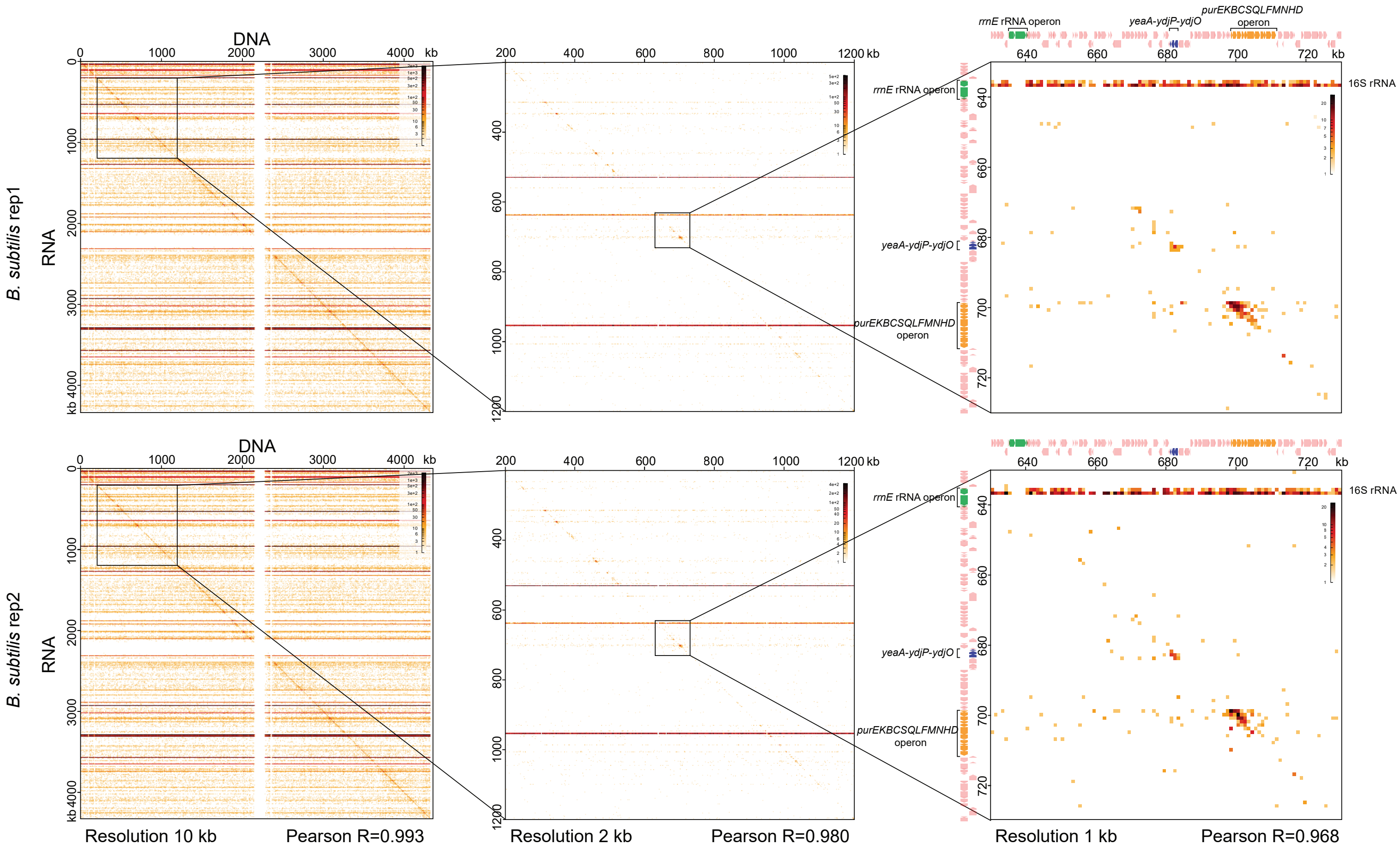

Supplementary Figure 5. RNA-DNA contact maps for biological replicates of the experiment with *B. subtilis*. All designations are as in Supplementary Fig. 4.

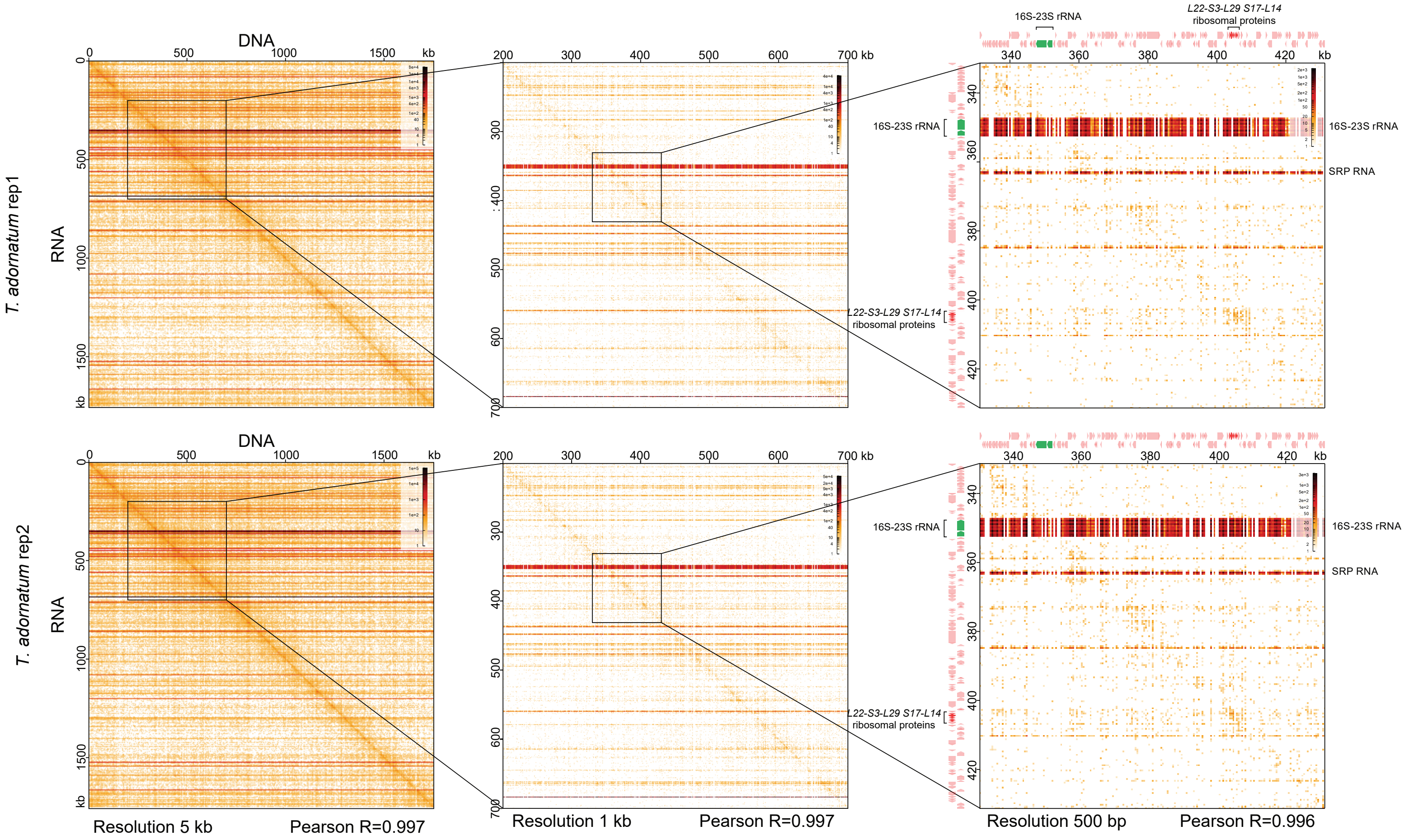

Supplementary Figure 6. RNA-DNA contact maps for biological replicates of the experiment with *T. adornatum*. All designations are as in Supplementary Fig. 4.

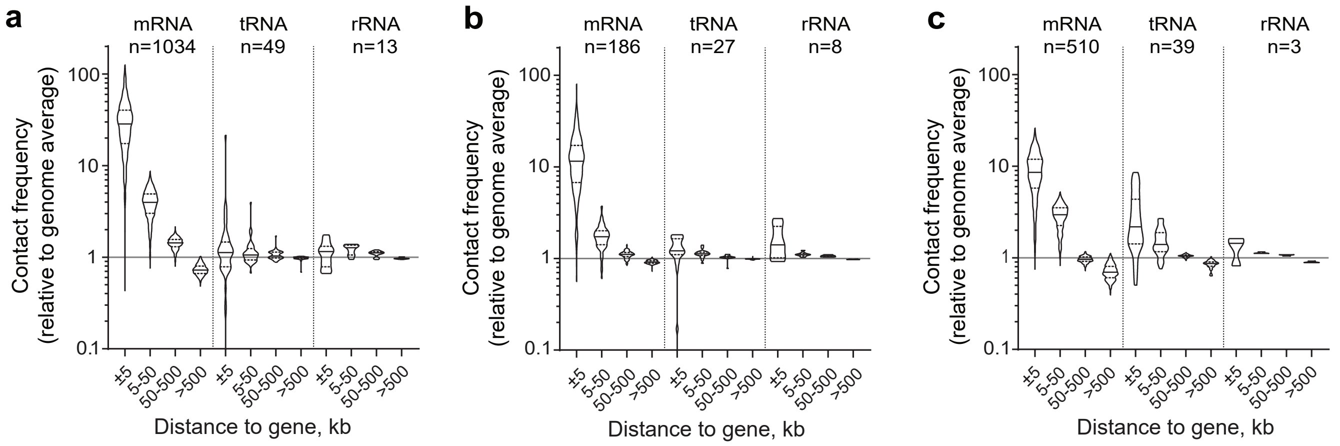

Supplementary Figure 7. Frequency of contacts of mRNAs, tRNAs and rRNAs in indicated genomic intervals for *E. coli* (a), *B. subtilis* (b) and *T. adornatum* (c) calculated without taking into consideretion the contacts with rRNA operons. The same as Fig. 1g-i, but the sequences of rRNA operons were excluded from all analyzed intervals and the interval lengths were adjusted accordingly. In the *T. adornatum* reference genome, 5S gene is situated ~330 kb away from 16S and 23S genes, so we excluded two regions (4742 bp region encompassing 16S and 23S genes and 116 bp region encompassing 5S gene).

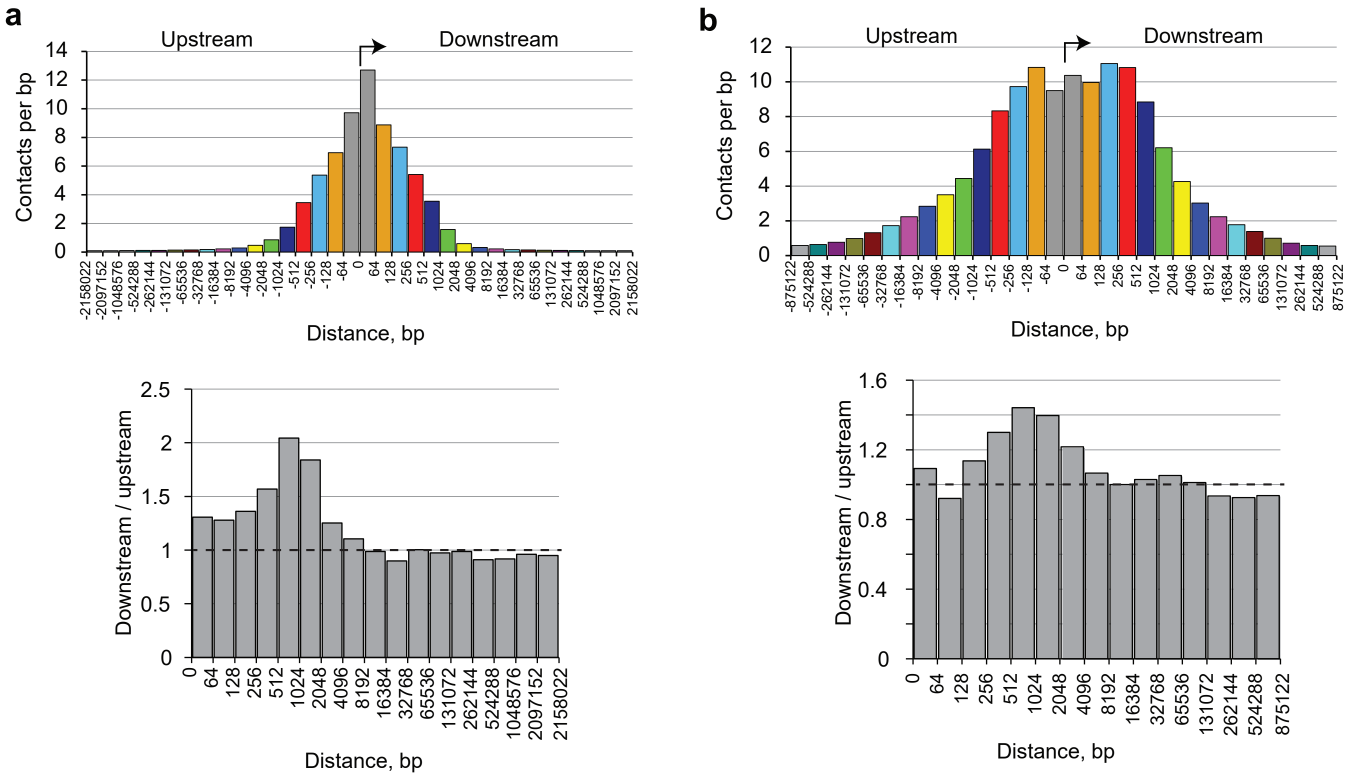

Supplementary Figure 8. Frequency of contacts of mRNA fragments in intervals upstream and downstream of the encoding DNA segment for *B. subtilis* (a) and *T. adornatum* (b). All designations are as in Fig. 2a.

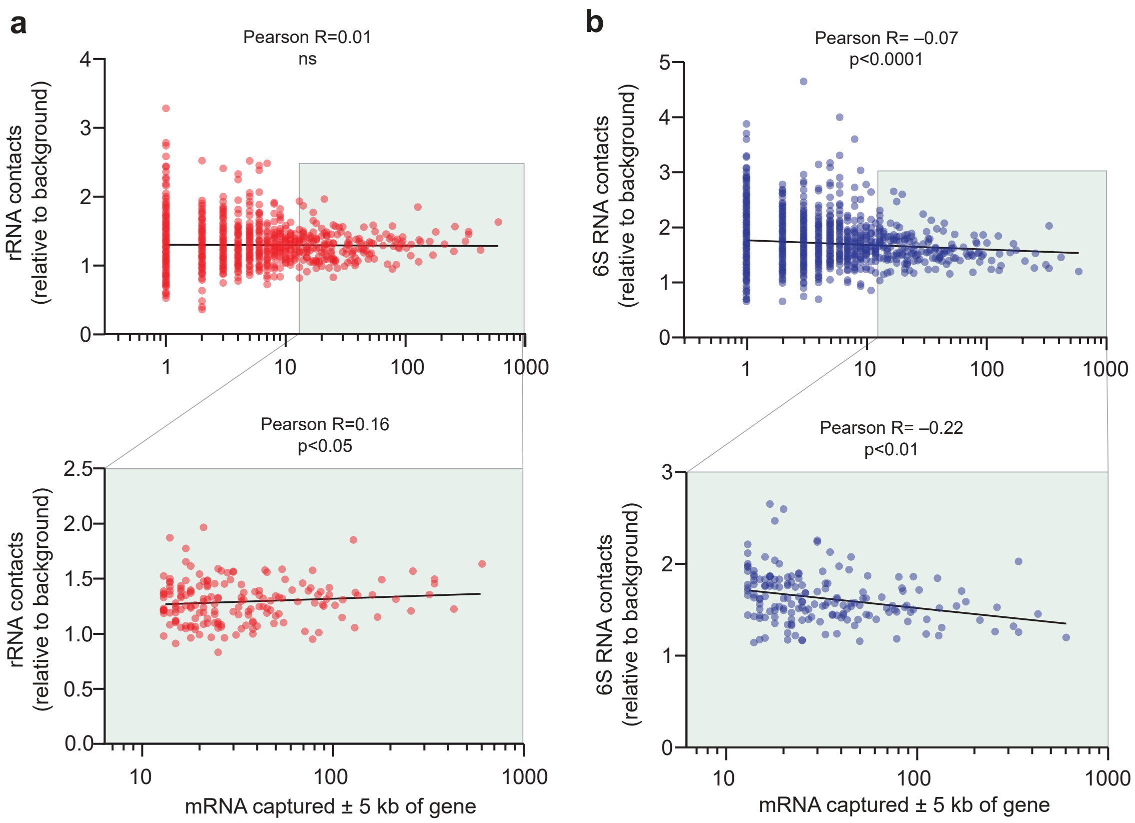

Supplementary Figure 9. Contacts of rRNA and 6S RNA with protein-coding genes in *B. subtilis*. All designations are as in Fig. 3.

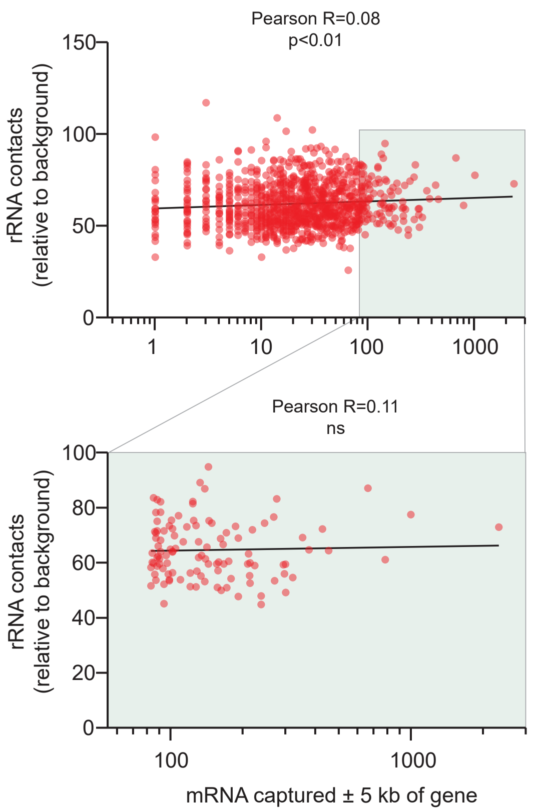

Supplementary Figure 10. Contacts of rRNA with protein-coding genes in *T. adornatum*. All designations are as in Fig. 3.

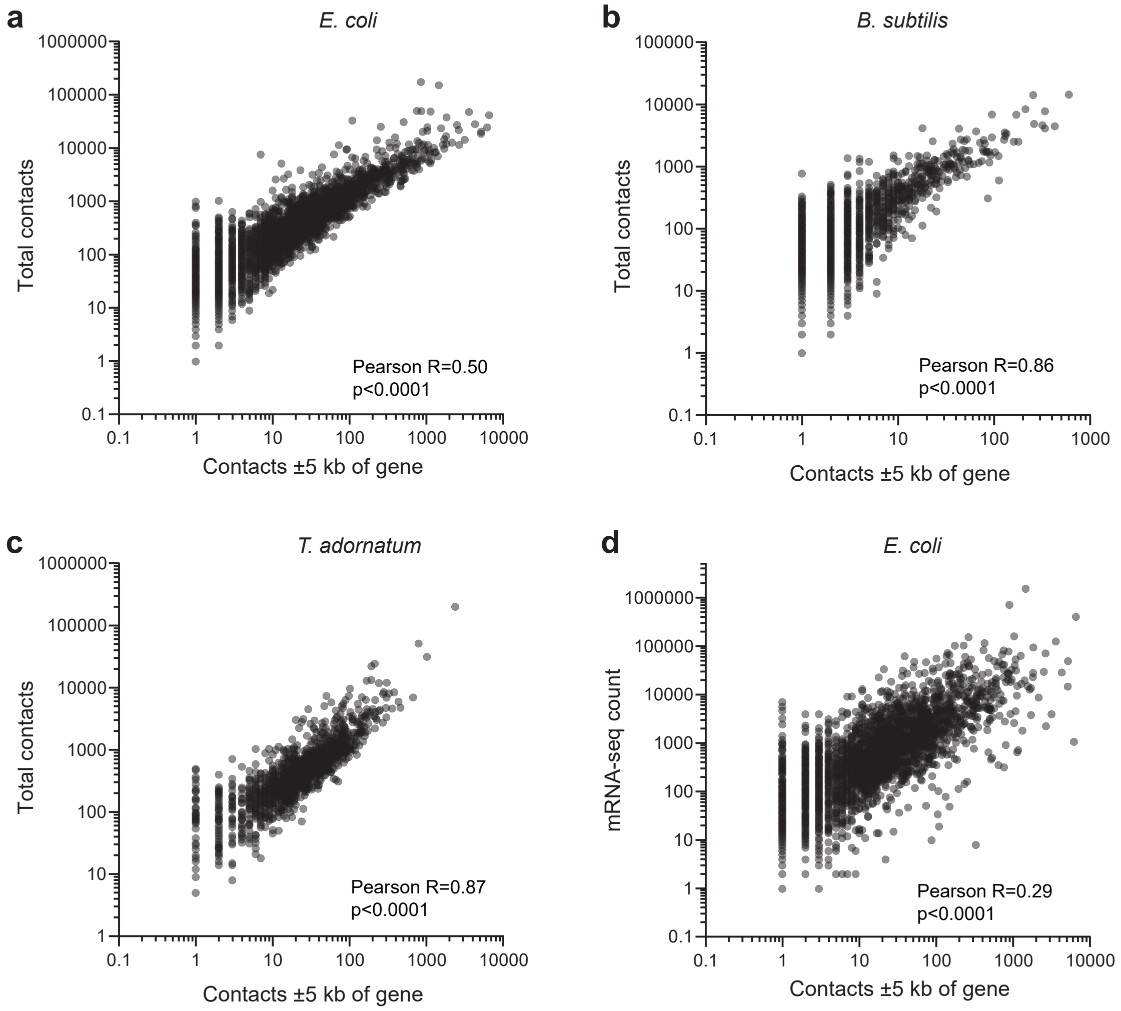

Supplementary Figure 11. Correlation between the number of mRNA contacts in the region ±5 kb of the middle of the encoding gene and the total number of mRNA contacts (a-c) or mRNA-seq signal (d). Scatter plots show the results for *E. coli* (**a, d**), *B. subtilis* (**b**) and *T. adornatum* (**c**).

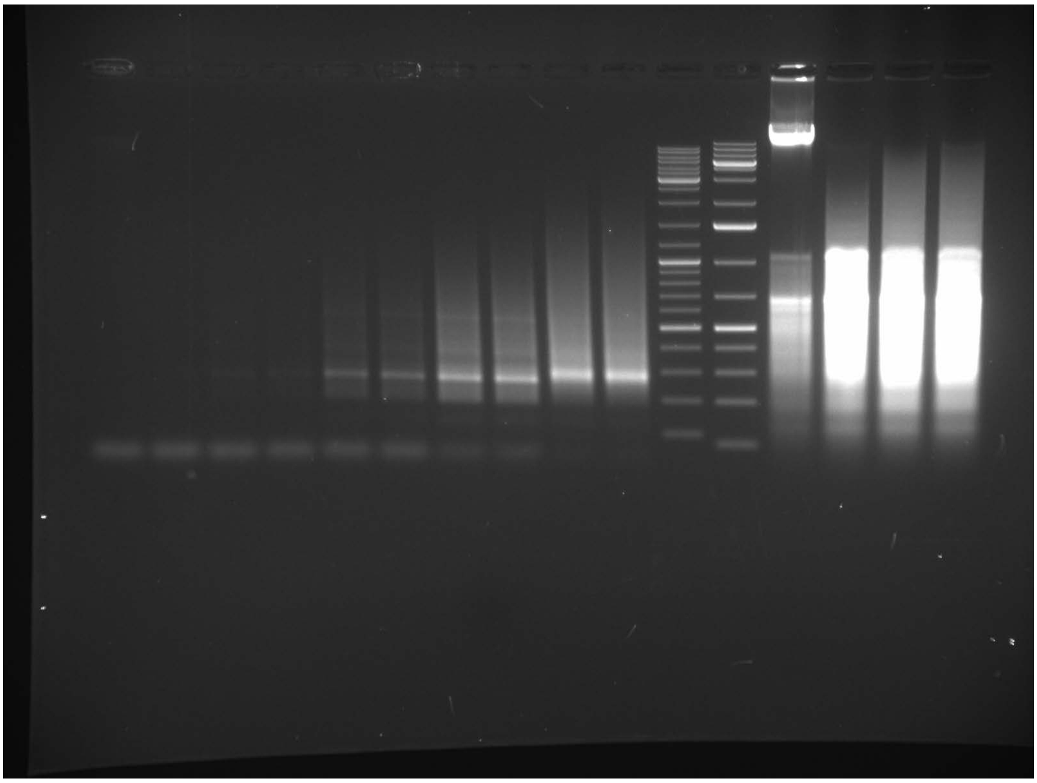

Supplementary Figure 12. The uncropped version of Supplementary Fig. 1.

Supplementary Tables

Supplementary Table 1. Statistics of read filtering and mapping.

Experiment	Raw read pairs	1: Bridge in direct read	2: Reverse read starts with GGG	3: DNA portion 18-20 nucleotides	4: RNA 3' and RNA 5' portions ≥ 10 nucleotides	5: All 3 portions passed trimming by quality	6: All 3 portions are uniquely mapped	7: RNA 3' and RNA 5' portions are mapped to opposite DNA strands	8: Distance between RNA 3' and RNA 5' portions < 10 kb
E. coli rep1	312,705,573	300,195,326	296,352,749	291,922,130	271,211,720	240,374,523	31,024,402	31,018,771	31,013,613
E. coli rep2	344,032,773	331,633,984	327,699,630	322,775,816	303,730,982	274,269,965	36,667,352	36,660,161	36,653,871
B. subtilis rep1	114,619,459	108,811,428	107,424,279	98,342,135	80,066,276	72,626,418	3,066,868	3,066,379	3,066,126
B. subtilis rep2	116,258,571	110,187,132	108,825,526	98,391,341	81,460,018	73,537,766	3,238,000	3,237,344	3,236,911
T. adornatum rep1	56,119,054	40,398,284	40,083,784	34,884,028	31,376,206	27,823,207	22,610,856	22,605,009	22,600,579
T. adornatum rep2	62,594,389	51,111,937	50,756,842	44,992,760	43,168,587	38,340,122	31,823,758	31,815,256	31,808,964

Supplementary Table 2. Representation of different RNA types in RNA-DNA interactomes of *E. coli*, *B. subtilis* and *T. adornatum*.

E. coli

RNA type	No of genes	No of contacts	Representation (%)
tRNA	85	23,649,617	34.9
6S RNA	1	10,992,421	16.2
SRP RNA	1	10,290,847	15.2
tmRNA	1	8,960,773	13.2
rRNA	22	6,020,248	8.9
protein coding	4,150	3,271,745	4.8
RNase P RNA	1	68,576	0.1
pseudogene	198	68,416	0.1
rprA RNA	1	1,810	<0.1
RtT RNA	6	336	<0.1
Unannotated		4,342,695	6.4

B. subtilis

RNA type	No of genes	No of contacts	Representation (%)
tRNA	86	3,626,247	57.5
6S	2	670,535	10.6
rRNA	30	494,967	7.9
protein coding	4,326	468,449	7.4
SRP RNA	1	460,958	7.3
tmRNA	1	368,844	5.9
pseudogene	103	9,043	0.1
RNase P RNA	1	2,163	<0.1
Unannotated		201,831	3.2

T. adornatum

RNA type	No of genes	No of contacts	Representation (%)
rRNA	3	44,876,314	82.5
protein coding	1,375	1,295,072	2.4
SRP RNA	1	460,958	0.8
tRNA	44	174,738	0.3
pseudogene	5	847	<0.1
Unannotated		7,601,614	3.2

Supplementary Table 3. Sequences of oligos and adapters used in RedC procedure.

Oligo/adapter	5'-3' sequence	Comment
ds bridge adapter (annealed from two oligos)	/rApp/TCCTAGCACCATCAATGCGATAGGCAACGCTCCGACT	3' hydroxyl non-blocked; underlined region, MmeI site
	/Phos/GTCGGAGCGTTGCC/T-Biotin/ATCG	underlined region, MmeI site
ds oligo with MmeI site (annealed from two oligos)	CTGTCCGTTCCGACTACCCTCCCGAC	underlined region, MmeI site
	GTCGGGAGGGTAGTCGGAACGGACAG	underlined region, MmeI site
switch template oligo	iCiGiCGTGACTGGAGTTCAGACGTGTGCTCTTCCGATCTrGrGrG	iC and iG, Iso-dC and Iso-dG; r, ribonucleotides
NN-adapter (annealed from two oligos)	AGATCGGAAGAGCGTCGTGTAGGGAAAGAGTGTAGATCTCGGTGGTCGCCGTATCATT	
	AATGATACGGCGACCACCGAGATCTACACTCTTTCCCTACACGACGCTCTTCCGATCTNN	N, any of 4 bases
PCR universal primer	AATGATACGGCGACCACCGAGATCTACACTCTTTCCCTACACGA	
PCR indexed primer (index 7)	CAAGCAGAAGACGGCATACGAGATGATCTGGTGACTGGAGTTCAGACGTGTGC	
PCR indexed primer (index 8)	CAAGCAGAAGACGGCATACGAGATTCAAGTGTGACTGGAGTTCAGACGTGTGC	
PCR indexed primer (index 24)	CAAGCAGAAGACGGCATACGAGATGCTACCGTGACTGGAGTTCAGACGTGTGC	
PCR indexed primer (index 26)	CAAGCAGAAGACGGCATACGAGATGCTCATGTGACTGGAGTTCAGACGTGTGC	
PCR indexed primer (index 27)	CAAGCAGAAGACGGCATACGAGATAGGAATGTGACTGGAGTTCAGACGTGTGC	
PCR indexed primer (index 31)	CAAGCAGAAGACGGCATACGAGATATCGTGGTGACTGGAGTTCAGACGTGTGC	
