# Supplementary material for: RNA-DNA interactomes of three prokaryotes uncovered by proximity ligation
Source: Commun Biol. 2023 Apr 29;6:473. doi: 10.1038/s42003-023-04853-8 (PMC10148824; doi:10.1038/s42003-023-04853-8)
Supplement: Supplementary file 3 — Description of Additional Supplementary Files [file 42003_2023_4853_MOESM3_ESM.pdf]

Description of Additional Supplementary Files

Supplementary Data 1. The number of RNA-DNA contacts identified for individual RNAs in experiments with *E. coli*. Columns A-G, characteristics of genes; columns H-J, number of contacts of respective RNAs in rep1, rep2, and in rep1+rep2; columns K-N, number of contacts of respective RNAs with genomic intervals; column O, P, number of contacts of rRNA and 6S RNA with respective genes; column Q, number of background contacts at respective genes; column R, mRNA-seq signal. Genes are ranked by the total number of contacts observed for respective RNAs (column J).

Supplementary Data 2. The number of RNA-DNA contacts identified for individual RNAs in experiments with *B. subtilis*. All designations are as in Supplementary Data 1.

Supplementary Data 3. The number of RNA-DNA contacts identified for individual RNAs in experiments with *T. adornatum*. All designations are as in Supplementary Data 1.

Supplementary Data 4. Numerical source data for Fig. 1g-i and Fig. 2a.
