# Supplementary material for: RNA-DNA interactomes of three prokaryotes uncovered by proximity ligation
Source: Commun Biol. 2023 Apr 29;6:473. doi: 10.1038/s42003-023-04853-8 (PMC10148824; doi:10.1038/s42003-023-04853-8)
Supplement: Supplementary file 1 — Razin_Peer Review File [file 42003_2023_4853_MOESM1_ESM.pdf]

nature portfolio

Peer Review File

RNA-DNA interactomes of three prokaryotes uncovered by proximity ligation.

Open Access This file is licensed under a Creative Commons Attribution 4.0 International License, which permits use, sharing, adaptation, distribution and reproduction in any medium or format, as long as you give appropriate credit to the original author(s) and the source, provide a link to the Creative Commons license, and indicate if changes were made. In the cases where the authors are anonymous, such as is the case for the reports of anonymous peer reviewers, author attribution should be to 'Anonymous Referee' followed by a clear attribution to the source work. The images or other third party material in this file are included in the article's Creative Commons license, unless indicated otherwise in a credit line to the material. If material is not included in the article's Creative Commons license and your intended use is not permitted by statutory regulation or exceeds the permitted use, you will need to obtain permission directly from the copyright holder. To view a copy of this license, visit http://creativecommons.org/licenses/by/4.0/.

Reviewers' comments:

Reviewer #1 (Remarks to the Author):

Review ms. no. COMMSBIO-22-3759-T

The authors investigated the RNA-DNA interactome of two bacteria (Escherichia coli, Bacillus subtilis) and the crenarchaeon Thermofilum adornatum by proximity ligation applying the RedC methodology. The authors infer from their data that mRNAs preferentially interact with their cognate genes and those downstream in the same polycistronic operon (consistent with mRNA 'hitchhiking' on the RNAP in the direction of transcription), (ii) rRNAs preferentially interact with active protein-coding genes in both bacteria and the archaeon (indicating co-transcriptional translation), and (iii) 6S RNAs are depleted from active genes in E. coli and B. subtilis.

Major comments:

1) p. 2, last line: the authors applied RedC to E. coli and B. subtilis in stationary phase where transcriptional activity decreases and and is changed relative to exponential growth. T. adornatum was analyzed in exponential growth phase. Explain this choice and discuss the issue in terms of comparability of results for the two bacteria and the archaeon, also regarding possible changes in the outcome of RedC analysis for the two bacteria when analyzed in exponential phase.

2) Fig. 1c-e, y-axis scale: if it represents the number of mapped RNA-DNA contacts, what means, for example, 0.4 in panel e?
Fig. 1g-i: lettering on the x-axes, replace <5 with ±5 (as in the text).

3) p. 3, lines 2/3 from bottom and Fig. 1i: tRNAs in T. adornatum showed some preference for the genomic region where they are encoded. This suggests to me that there was active tRNA transcription during exponential growth phase in the archaeon, but not in the two bacteria during stationary phase. This point should be discussed.

4) Fig. S3: the lettering within the figure panels is hard to decipher, should be increased; "triangles near diagonal and horizontal lines" remains unclear when inspecting Fig. S3; I only see a triangle pattern at the aceBAK operon in the panels on the right. Improve guiding of the reader by adding more information to the figure legend. Also add a sentence to the legend, in the sense "For the lack of rRNA-DNA contacts in rRNA operons, see text", as the reader is puzzled by this finding when initially (on p. 3, line 17) referred to Fig. S3.

5) p. 3, line 2 from bottom, to line 2 on p. 4: how did you map rRNA-DNA reads to the 7 and 10 rRNA operons in E. coli and B. subtilis? Did you assign 1/7 or 1/10 of the contacts to each rRNA operon? Could it be that the ceased rRNA transcription during stationary phase has contributed to these white areas at rRNA operons in Fig. S3 and S4? This possibility would be consistent with the presence of rRNA-DNA contacts at the rRNA operon of T. adornatum that is actively transcribed during exponential phase. This aspect should also be considered in the text on p. 4, last paragraph; there, I do not find the explanation convincing ("The absence of enrichment at the parental gene may reflect the high stability of tRNA and rRNA molecules and their rapid release from the parental gene upon transcription termination and diffusion to the place of functioning.").

6) p. 4, 2nd paragraph, Fig. S6a: at short distances a higher frequency of contacts with downstream intervals versus equally spaced upstream intervals was observed, but the difference between downstream and upstream contact frequencies disappeared at a distance > 2 kb. These findings are not clear to me. As mRNA 3'-ends are ligated to the bridge adapter and RNA 3'-ends are present in the active site of RNAP, I would not expect any contact ligation to upstream regions of the polycistronic operon that encodes the mRNA. How to explain? May mRNA fragmentation occur at any step of the procedure before bridge adapter ligation? Please discuss.

7) p. 4, last two lines, "To account for the differences in DNA accessibility and the number of restriction sites for different genes ..." It is unclear what you mean here, please rephrase.

8) Figs. 3, S7 and S8: I have difficulties to accept that that there is a robust positive correlation between the number of rRNA contacts and the transcriptional activity when considering all protein-coding genes, as illustrated in the upper panels of Figs. 3a, S7a and S8. For example, in Fig. 3a (upper panel), there is large fluctuation of rRNA contact numbers particularly for weakly expressed mRNAs (x-axis range 1 to ~100) and I see many loci with increased rRNA contacts; could this observation mean that the dwell time of ribosomes is prolonged at many weakly expressed protein genes owing to the presence of rare codons?
Also for 6S RNA, although there might be a weak trend toward a decrease of 6S RNAs at actively transcribed genes, I see as key feature a substantial scattering of 6S RNA presence at protein-coding genes. This observation is consistent with the surprisingly selective inhibition of genes by 6S RNAs in the two bacteria. In Fig. 8 (lower part), the scattering of data points and the low number of data points at > 200 reads make the prediction of a clear trend questionable.
Taken together, I propose to interpret and discuss these findings more cautiously.

9) p. 5, line 7 from bottom: please mention which fraction of rRNA contacts are assignable to B. subtilis 6S-1 and 6S-2 RNA.

10) Table S1: explain the background of "7: RNA 3' and RNA 5' portions are mapped to opposite DNA strands" and "8: Distance between RNA 3' and RNA 5' portions < 10 kb" to the general reader.

11) Table S3: mention that the ds bridge adapter is annealed from two oligos; mark the MmeI sites in the ds bridge adapter and in the ds oligo with MmeI site; explain that there is a MmeI recognition site in the ds bridge adapter to uniformly generate DNA portions of 18-20 nt.

12) p. 9, last line: what are those GGG/CCC oligonucleotides?

13) p. 10, lines 7/8: what is meant by "Before mapping, the end of the DNA portion adjoining the bridge was supplemented with CATG ..." ?

Minor comments:

Generally, when referring to figures in the text, write, for example, "Fig. 2a, upper panel" or "Fig. 2a, upper part" instead of "Fig. 2a, upper"

p. 1:
- Abstract, line 4, rewrite: "... genomes of E. coli, B. subtilis and the thermophilic archaeon T. adornatum."

p. 2:
- 1st line: write out "TADs" when first mentioned;
- line 15: "... RNA-DNA interactomes of two bacteria and one archaeon. We ..."
- line 18: "... that in bacteria, noncoding 6S RNAs are depleted ... which is consistent with their negative role"
- last line: archaeon (not archaea); delete "exotic"

p. 3:
- line 6: add here that E. coli has 7 and B. subtilis 10 rRNA operons;
- line 11: " Notably, the number of RNA-DNA contacts for a particular mRNA is ..."

p. 4:
- line 1: replace "lines" with "areas"

p. 18:
- legend to Fig. 3, line 3: rewrite "within 5 kb of the middle of the gene" to "within ±5 kb from the middle of the gene"

Reviewer #2 (Remarks to the Author):

In this work Gavrilov et al. performed Red-C in two bacteria and one archaea mapping the RNA-DNA interactions. The group previously published Red-C technology. The authors found that mRNA are located at their transcribed regions but ribosomal RNAs and tRNAs interact with active genes. Furthermore, negative transcription regulator 6s noncoding RNA are depleted from active genes. A notable concept is that in bacteria and archaea, co-transcriptional translation is common; but in eukaryote, ribosomal RNA does not bind to active genes. Overall, this study clearly has its value by providing maps in new organisms. The paper is well written and easy to follow. I think it is a good candidate for Communications Biology. I have a few comments or questions.

1. In the RNA-DNA heatmap like Fig. 1c-e, the authors showed that rRNA, tRNA, form horizontal lines. But Fig. 1b also shows other RNAs, including pseudogenes and some protein coding RNAs also form many contacts, perhaps forming horizontal lines. Can the authors provide more information on what those genes are? Are there any explanations or discussions?

2. It seems that the RNA-DNA heatmaps also have some vertical lines. Do they indicate that some genomic region attracts many RNAs? Are these vertical lines protein coding genes? Can the authors come up with some ranking systems for these locations? It will be nice to have more discussion on those.

3. Fig 3 shows a positive correlation between rRNA contacts to gene express levels. However, in the context of co-transcriptional translation, it will be more interesting to ask if rRNA contacts are correlated with translation rate. Can the authors explore or discuss this question?

Reviewer #3 (Remarks to the Author):

This is a nice and focused study by Gavrilov and colleagues. Although they did not discover any "extraordinary" facts in the bacterial DNA-RNA interactome, their results confirm the applicability of RedC methods to study DNA-RNA interactions in different prokaryotic organisms. I suggest the manuscript to be published in its current form.

Point-by-point response to the reviewers' comments

Reviewer 1

Comment

1) p. 2, last line: the authors applied RedC to E. coli and B. subtilis in stationary phase where transcriptional activity decreases and and is changed relative to exponential growth. T. adornatum was analyzed in exponential growth phase. Explain this choice and discuss the issue in terms of comparability of results for the two bacteria and the archaeon, also regarding possible changes in the outcome of RedC analysis for the two bacteria when analyzed in exponential phase.

Answer

We rewrote the third sentence of the Results and Discussion section in the following way:

"We applied RedC to classical laboratory bacteria *Escherichia coli* and *Bacillus subtilis* in stationary growth phase, as well as to the thermophilic archaeon *Thermofilum adornatum*[19] in exponential growth phase, to test the applicability of the protocol for mapping RNA-DNA interactions under various experimental conditions".

Hereafter, where necessary, we interpret results in the context of growth phase. In the description of tRNA genomic distribution, we note: "The slight enrichment at the parental locus for tRNAs in *T. adornatum* may be due to active tRNA transcription during exponential growth of this archaeon". In the legend to Fig. S3, we note: "The ceased rRNA transcription during stationary phase may also contribute to horizontal white areas at rRNA operons".

Comment

2) Fig. 1c-e, y-axis scale: if it represents the number of mapped RNA-DNA contacts, what means, for example, 0.4 in panel e?

Fig. 1g-i: lettering on the x-axes, replace <5 with ±5 (as in the text).

Answer

The scale of x and y axes of contact maps represents genomic coordinates. In Fig. 1c-e, the scale is in Mb, so 0.4 in panel e means 0.4 Mb. In the original version of the MS, we indicated the units of measurement only along the x-axes of contact maps. In the revised version, we indicated units of measurement (symbols "Mb" or "kb") along the y-axes as well.

The number of contacts is represented by color intensity according to the scale shown to the right of each map. We enlarged the lettering along color scales in the revised version of the MS.

We replaced <5 with ±5 as suggested by the reviewer.

Comment

3) p. 3, lines 2/3 from bottom and Fig. 1i: tRNAs in T. adornatum showed some preference for the genomic region where they are encoded. This suggests to me that there was active tRNA transcription during exponential growth phase in the archaeon, but not in the two bacteria during stationary phase. This point should be discussed.

Answer

The following sentence was added to the text: “The slight enrichment of tRNAs at their parental loci in *T. adornatum* may be due to active tRNA transcription during exponential growth of this archaeon”.

Comment

4) Fig. S3: the lettering within the figure panels is hard to decipher, should be increased; "triangles near diagonal and horizontal lines" remains unclear when inspecting Fig. S3; I only see a triangle pattern at the aceBAK operon in the panels on the right. Improve guiding of the reader by adding more information to the figure legend. Also add a sentence to the legend, in the sense "For the lack of rRNA-DNA contacts in rRNA operons, see text", as the reader is puzzled by this finding when initially (on p. 3, line 17) referred to Fig. S3.

Answer

We increased the lettering within the figure panels and added more information in the figure legend:

“Positions of genes and operons are indicated on high-resolution maps (maps on the right). Note characteristic contact patterns on the right maps: triangles near diagonal at the aceBAK operon and the rplKAJL-rpoBC operon; horizontal lines at the Thr-Tyr-Gly-Thr tRNA gene cluster and the 16S and 5S rRNA genes of the rrnB rRNA operon. White horizontal areas occupying the major portion of the rrnB rRNA operon and the whole rrnE rRNA operon are due to the lack of uniquely mapped RNA reads in these genomic regions (see the text)”.

Comment

5) p. 3, line 2 from bottom, to line 2 on p. 4: how did you map rRNA-DNA reads to the 7 and 10 rRNA operons in E. coli and B. subtilis? Did you assign 1/7 or 1/10 of the contacts to each rRNA operon? Could it be that the ceased rRNA transcription during stationary phase has contributed to these white areas at rRNA operons in Fig. S3 and S4? This possibility would be consistent with the presence of rRNA-DNA contacts at the rRNA operon of T. adornatum that is actively transcribed during exponential phase. This aspect should also be considered in the text on p. 4, last paragraph; there, I do not find the explanation convincing ("The absence of enrichment at the parental gene may reflect the high stability of tRNA and rRNA molecules and their rapid release from the parental gene upon transcription termination and diffusion to the place of functioning.").

Answer

As noted in the beginning of the Results and Discussion section, we work with unique RNA-DNA contacts:

"For two biological replicates of experiments with *E. coli, B. subtilis,* and *T. adornatum*, 656M, 230M, and 118M paired-end reads were obtained, respectively, yielding 67M, 6M, and 54M unique RNA-DNA contacts (Supplementary Table 1)".

In the "Read filtering and mapping" section of Methods it is specified that "We retained only such DNA-RNA 3'-RNA 5' triples that were all successfully and uniquely mapped to the reference genome. If one of the portions was missing, non-uniquely mapped, or unmapped, the read pair was filtered out".

So, we do not assign 1/7 or 1/10 of the contacts to each rRNA operon. We work with contacts that were unambiguously assigned to particular rRNAs. The contact numbers for individual RNAs can be found in excel spreadsheet Supplementary Data 1 (E. coli), Supplementary Data 2 (B. subtilis), and Supplementary Data 3 (T. adornatum). Here is an output for rRNAs in E.coli:

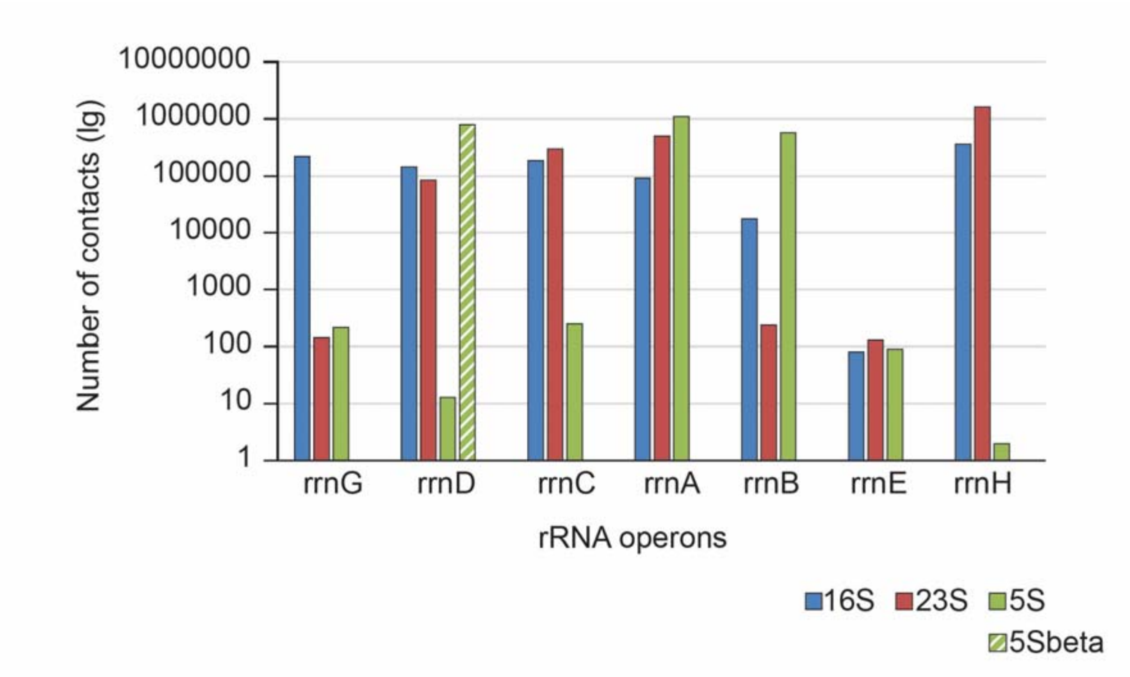

It is seen that the number of mapped (unique) contacts for different rRNAs belonging to the same operon and for different operons varies drastically. Inspection of RNA-DNA contacts maps also shows a huge variation in the number of contacts identified for different portions of the same rRNA molecule. For example, Supplementary Figure 3 shows the contacts of 16S rRNA from the rrnB rRNA operon. It can be seen that the contacts are only detected for the end of 16S rRNA. In view of such variations in contact numbers caused by multiple mapping issues, it is difficult to consider the absence or small number of contacts of some rRNAs as an indication of low transcription level of the corresponding operon. With that said, we do agree with the reviewer that the ceased rRNA transcription during stationary phase also may contribute to white areas at rRNA operons in RNA-DNA contact maps.

To explain the mapping procedure and how it influences the number of identified rRNA contacts, the following sentence was added to the first paragraph of the Results and Discussion sentence:

"In contrast, the presence of 7 and 10 rRNA operons with a similar sequence in the genomes of *E. coli* and *B. subtilis*, respectively, sharply reduces the possibility of unambiguous mapping of rRNA fragments".

We also added the following sentences in the legend to Supplementary Figure 3:

"White horizontal areas occupying the major portion of the rrnB rRNA operon and the whole rrnE rRNA operon are due to the lack of uniquely mapped RNA reads in these genomic regions

(see the text). The ceased rRNA transcription during stationary phase may also contribute to horizontal white areas at rRNA operons".

Despite the fact that we identify only a fraction of rRNA contacts (and possible a very small fraction for some RNAs), based on this fraction it is still possible to assess the distribution of rRNAs along the genome, provided there are enough counts for statistically meaningful analysis. However, the poor read mappability along rRNA operons should again be taken into account when interpreting the distribution of rRNA contacts in the vicinity of the parental locus. Indeed, the used mapping procedure filters out not only many rRNA-DNA ligation products representing contacts of rRNA with different genomic regions, but also many RNA-rDNA ligation products representing contacts of different RNAs (including rRNA itself) with rRNA operons. As a result, we observe an approximately two-fold reduction in the frequency of contacts of rRNA within a 10 kb region harboring the parental operon relative to the frequency of rRNA contacts in more remote genomic regions (Fig. 1g, h). The degree of reduction (two-fold) corresponds to the portion of the 10 kb region occupied by the rRNA operon (~ 5 kb).

The decreased number of rRNA contacts at the parental locus is discussed in the following sentence, which was slightly modified compared to the original version of the MS:

"The apparent depletion of rRNAs from their parental loci in *E. coli* and *B. subtilis* (Fig. 1g, h) is due to a poor mappability of DNA reads along the multi-copy rRNA operons, which leads to the appearance of vertical white areas in the contact map (clearly visible in Supplementary Fig. 3)".

We further note in the legend to Supplementary Figure 3:

"Also note white vertical areas at the both rRNA operons, indicating the absence of uniquely mapped DNA reads in these genomic regions".

Comment

6) p. 4, 2nd paragraph, Fig. S6a: at short distances a higher frequency of contacts with downstream intervals versus equally spaced upstream intervals was observed, but the difference between downstream and upstream contact frequencies disappeared at a distance > 2 kb. These findings are not clear to me. As mRNA 3'-ends are ligated to the bridge adapter and RNA 3'-ends are present in the active site of RNAP, I would not expect any contact ligation to upstream regions of the polycistronic operon that encodes the mRNA. How to explain? May mRNA fragmentation occur at any step of the procedure before bridge adapter ligation? Please discuss.

Answer

The following sentence was added to the paragraph in question:

“Occasional fragmentation of RNA before bridge adapter ligation makes it possible to analyze the distribution of nascent transcripts along the transcribed area when transcription is not yet finished and the 3’ end of the RNA is in the active site of RNA polymerase”.

We also extended the legend to Supplementary Figure 1 presenting the results of gel electrophoresis of products of amplification of RNA-DNA chimeras to acknowledge RNA fragmentation in the procedure:

“Short RNA portions may represent short RNA species or originate as a result of occasional fragmentation of RNA during experimental procedure”.

Comment

7) p. 4, last two lines, "To account for the differences in DNA accessibility and the number of restriction sites for different genes ..." It is unclear what you mean here, please rephrase.

Answer

The text was rephrased to:

“For correct analysis, we had to take into consideration that the number of contacts determined for different DNA regions may vary depending on technical factors such as the efficiency of cross-linking and restriction enzyme digestion, as well as DNA read mappability at a given genomic location. Moreover, longer genes were expected to produce more ligation products with any RNA than shorter ones. To account for differences in the gene length and the efficiency of the RedC procedure for different DNA regions …”

Comment

8) Figs. 3, S7 and S8: I have difficulties to accept that that there is a robust positive correlation between the number of rRNA contacts and the transcriptional activity when considering all protein-coding genes, as illustrated in the upper panels of Figs. 3a, S7a and S8. For example, in Fig. 3a (upper panel), there is large fluctuation of rRNA contact numbers particularly for weakly expressed mRNAs (x-axis range 1 to ~100) and I see many loci with increased rRNA contacts; could this observation mean that the dwell time of ribosomes is prolonged at many weakly expressed protein genes owing to the presence of rare codons?

Also for 6S RNA, although there might be a weak trend toward a decrease of 6S RNAs at actively transcribed genes, I see as key feature a substantial scattering of 6S RNA presence at protein-coding genes. This observation is consistent with the surprisingly selective inhibition of genes by 6S RNAs in the two bacteria. In Fig. 8 (lower part), the scattering of data points and the low number of data points at > 200 reads make the prediction of a clear trend questionable.

Taken together, I propose to interpret and discuss these findings more cautiously.

Answer

We tried to soften the conclusions of the section. "The correlation is especially evident…" was replaced with "The correlation is more evident…". We acknowledged fluctuation of rRNA contacts at weakly expressed protein-coding genes and discussed this observation as suggested by the reviewer: "At the same time, large fluctuations of rRNA contact numbers observed for weakly expressed protein-coding genes could indicate the presence of stalled ribosomes". Throughout the section we discuss only statistically significant trends (in order to emphasize this circumstance, we used the expression "weak, but significant correlation….").

Comment

9) p. 5, line 7 from bottom: please mention which fraction of rRNA contacts are assignable to B. subtilis 6S-1 and 6S-2 RNA.

Answer

If we understood the reviewer correctly, the reviewer is interested to know if some of the contacts assigned to rRNA can also be assigned to 6S-1 and 6S-2 RNAs in B. subtilis. We couldn't find similarity between the sequences of rRNA and 6S RNA genes using the BLAST alignment tool. Even if an RNA portion of RNA-DNA ligation product could occasionally map to both rRNA and 6S RNA genes (creating uncertainty about the gene this RNA portion originates from), the mapping algorithm would filter out such RNA-DNA contact because of the multiple mapping.

The reviewer might also mean "which fraction of rRNA contacts are assignable to B. subtilis 6S-1 and 6S-2 RNA *genes*". In our analysis (Fig. 3a, S7a and S8) we considered rRNA contacts with protein-coding genes. The number of rRNA contacts with 6S-1 and 6S-2 genes (and every other gene of the genome) can be found in Supplementary Data 2. It constitutes 97 and 136 for 6S-1 and 6S-2 genes, respectively, which is a minute fraction of all rRNA contacts in the genome (494967). If we normalize the number of rRNA contacts by the number of background

contacts at 6S-1 and 6S-2 genes (92 and 116, respectively, Supplementary Data 2), we get 1.05 and 1.17 background normalized rRNA contacts at 6S-1 and 6S-2 genes.

Here is how Fig. S7a would look if we included 6S-1 and 6S-2 genes in the analysis:

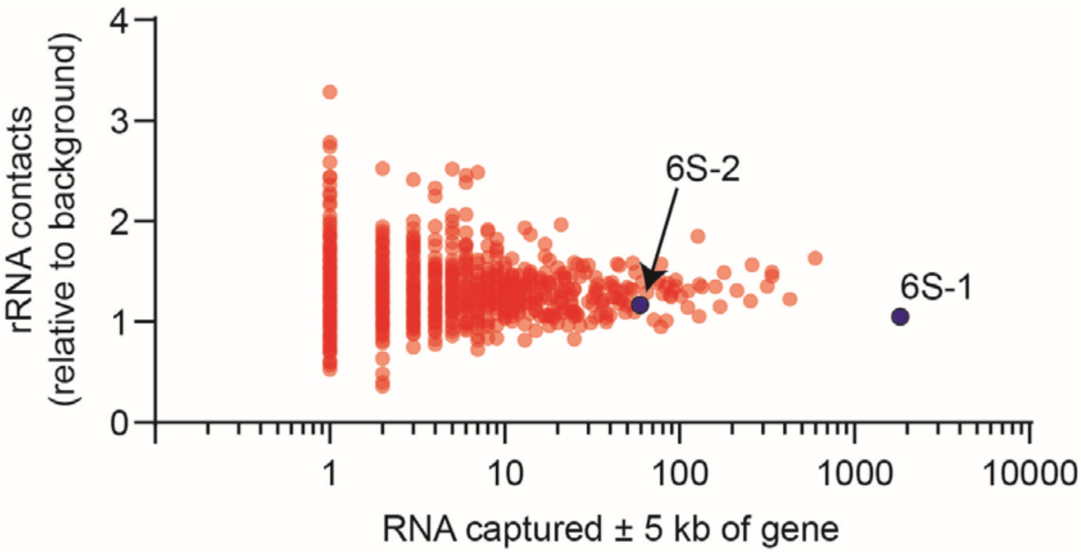

Comment

10) Table S1: explain the background of "7: RNA 3' and RNA 5' portions are mapped to opposite DNA strands" and "8: Distance between RNA 3' and RNA 5' portions < 10 kb" to the general reader.

Answer

In the end of the “Read filtering and mapping” section of Methods, we replaced “Finally, filters for random template switching of reverse transcriptase were applied” by “Finally, to avoid artifacts of intermolecular template switching of the reverse transcriptase, we discarded cases when RNA 3’ and RNA 5’ portions were mapped far apart from each other (>10 Kb) or to the same DNA strand”.

Comment

11) Table S3: mention that the ds bridge adapter is annealed from two oligos; mark the MmeI sites in the ds bridge adapter and in the ds oligo with MmeI site; explain that there is a MmeI recognition site in the ds bridge adapter to uniformly generate DNA portions of 18-20 nt.

Answer

In the revised version of Table S3 we indicated that adapters are annealed from two oligos and underlined the MmeI site in the sequence of the oligos. In the "RedC procedure" section of Methods, within the paragraph describing MmeI digestion, we added: "The recognition site of MmeI is incorporated into the bridge adapter to uniformly generate DNA portions of 18-20 nt. A short ds oligo containing the MmeI recognition site is added to stimulate the cleavage of DNA molecules containing a single MmeI site". The reader can also refer to our original article introducing the RedC technology (ref. 16) where the procedure is described in detail.

Comment

12) p. 9, last line: what are those GGG/CCC oligonucleotides?

Answer

GGG/CCC relates to the template switching during reverse transcription. We added the following sentences to the paragraph of Methods describing reverse transcription: "Reverse transcriptase first transcribes bridge DNA, then DNA-RNA junction, then RNA. Upon reaching the 5' end of the RNA, reverse transcriptase adds a few non-template nucleotides (predominantly dC) to the 3' end of cDNA. This dC stretch pairs with rGrGrG of the switch template oligo, and reverse transcriptase continues replication using the switch template oligo as a template (SMART technology). The switch template oligo provides a site for annealing of the Illumina indexed primer[16]". Taking into account this explanation, it should be clear what is meant by GGG/CCC oligonucleotides further in the text. We also added a sentence explaining the purpose of the NN-adapter ligation: "The NN-adapter provides a site for annealing of the Illumina universal primer[16]".

Comment

13) p. 10, lines 7/8: what is meant by "Before mapping, the end of the DNA portion adjoining the bridge was supplemented with CATG ..." ?

Answer

The section was rewritten in the following way:

"DNA portions of 18-20 nucleotides, RNA 3' portions of $\geq$ 10 nucleotides, and RNA 5' portions of $\geq$ 10 nucleotides were selected for the next mapping step. Before mapping, the end of the

DNA portion adjoining the bridge was supplemented with CATG (the 3' overhang produced by NlaIII digestion and then blunted) to reestablish the original sequence. "Reattaching" CATG to DNA portions increases their length to 22-24 nucleotides, which improves the yield of unique mappings".

Comment

Generally, when referring to figures in the text, write, for example, "Fig. 2a, upper panel" or "Fig. 2a, upper part" instead of "Fig. 2a, upper"

Answer

Rewritten as suggested by the reviewer.

Comment

p. 1:

- Abstract, line 4, rewrite: "... genomes of E. coli, B. subtilis and the thermophilic archaeon T. adornatum."

Answer

Done.

Comment

p. 2:

- 1st line: write out "TADs" when first mentioned;

- line 15: "... RNA-DNA interactomes of two bacteria and one archaeon. We ..."

- line 18: "... that in bacteria, noncoding 6S RNAs are depleted ... which is consistent with their negative role"

- last line: archaeon (not archaea); delete "exotic"

Answer

Done.

Comment

p. 3:

- line 6: add here that E. coli has 7 and B. subtilis 10 rRNA operons;

- line 11: " Notably, the number of RNA-DNA contacts for a particular mRNA is ..."

Answer

Done.

Comment

p. 4:

- line 1: replace "lines" with "areas"

Answer

Done.

Comment

p. 18:

- legend to Fig. 3, line 3: rewrite "within 5 kb of the middle of the gene" to "within ±5 kb from the middle of the gene"

Answer

Done.

Reviewer 2

Comment

1. In the RNA-DNA heatmap like Fig. 1c-e, the authors showed that rRNA, tRNA, form horizontal lines. But Fig. 1b also shows other RNAs, including pseudogenes and some protein coding RNAs also form many contacts, perhaps forming horizontal lines. Can the authors provide more information on what those genes are? Are there any explanations or discussions?

Answer

After discussion of the Fig. 1b results, we added the following sentence:

“Previous studies on RNA-DNA interactions in eukaryotes have also reported a correlation between the level of transcript and the number of contacts it makes in the genome[13-16]”.

Further in the text, we provided more discussion on the horizontal lines observed in RNA-DNA contacts maps:

“mRNAs expressed at high levels may also form horizontal lines, probably reflecting nonspecific contacts of mRNAs along the genome, however these lines almost always show increased intensity near the diagonal whereas the lines formed by tRNAs and rRNAs do not”.

Comment

2. It seems that the RNA-DNA heatmaps also have some vertical lines. Do they indicate that some genomic region attracts many RNAs? Are these vertical lines protein coding genes? Can the authors come up with some ranking systems for these locations? It will be nice to have more discussion on those.

Answer

Vertical lines are more visible in low resolution contact maps demonstrating RNA-DNA contacts for the whole genome (Fig. 1c-e, left panels in Supplementary Figs 3-5). These lines cover large genomic areas (have a width of up to several tens Kb) and become diffused and poorly distinguishable in higher resolution maps. We found that vertical lines frequently appear at genomic regions flanked by white vertical columns, which represent genomic regions showing little or no contacts with any RNA. The decreased number of RNA contacts at a particular genomic region may result from the poor mappability of DNA reads at this region or from the lack of NlaIII digestion sites. The later notion is well demonstrated by the contact maps of the highest resolution (100 bp, Fig. 2b, d). These map look all streaked due to the absence of NlaIII sites in many 100 bp bins. To clarify that the vertical lines seen in Fig. 2b,d do not reflect biologically relevant effects, the following sentence was added to the Fig. 2 legend:

“Note that a vertical streaked pattern in contact maps in (**b**) and (**d**) is due to the absence of NlaIII sites in many genomic bins at used map resolution (100 bp)”.

We also discussed the technical factors that can influence the number of identified RNA-DNA contacts in a particular genomic region in the following context:

“For correct analysis, we had to take into consideration that the number of contacts determined for different DNA regions may vary depending on technical factors such as region-specific efficiency of cross-linking and restriction enzyme digestion, as well as DNA read mappability at

a given genomic location. Moreover, longer genes were expected to produce more ligation products with any RNA than shorter ones. To account for differences in the gene length and the efficiency of the RedC procedure for different DNA regions,…”

Overall, the question regarding the nature of what is perceived at various scales as “vertical lines” requires further investigation. Although we do believe that some vertical lines represent genomic region attracting many RNAs, the identification of those would require accurate algorithms of analysis that would account for all the technical issues discussed above.

Comment

3. Fig 3 shows a positive correlation between rRNA contacts to gene express levels. However, in the context of co-transcriptional translation, it will be more interesting to ask if rRNA contacts are correlated with translation rate. Can the authors explore or discuss this question?

Answer

We haven’t analyzed correlation between rRNA contacts and translation rate in the current study. We discussed this idea in the following sentence:

“As a direction for future research, it would be interesting to explore if the contacts of rRNA with protein-coding genes are also correlated with the translational rate of the corresponding mRNAs”.

Reviewer 3

No comments to address.

Reviewers' comments:

Reviewer #2 (Remarks to the Author):

The authors have addressed all my comments.

As requested by the editor, I have also read the authors' responses to reviewer #1. In my opinion the authors have fully address all the comments by reviewer #1 as well.

Point-by-point response to the critical comments

Comment

#1- Given that we logically expect to have more rRNAs close to loci with intense mRNA transcription, isn't it surprising that the correlation is weak in the observed data? A few lines on this would be welcome. For instance, (Nevo-Dinur et al., 2011) found that mRNAs may localize more with the intended localization of the protein, rather than with the gene loci; other studies also found that mRNA did not primarily localize at their gene loci (dos Santos et al., 2012, Dugar et al., 2016, Fei et al., 2015, Kawamoto et al., 2005, Pilhofer et al., 2009); or that for membrane proteins, the mRNAs would localize to membranes (Moffitt et al., 2016) or that mRNAs from sets of genes from particular chromosome regions did not localize close to that region (Moffitt et al., 2016). These could explain the relatively weak correlations, nevertheless, your data still suggests some statistically significant correlation overall.

K. Nevo-Dinur, A. Nussbaum-Shochat, S. Ben-Yehuda, O. Amster-Choder (2011) Translation-independent localization of mRNA in E. coli. Science, 331 (2011), pp. 1081-1084

M. Pilhofer, M. Pavlekovic, N.M. Lee, W. Ludwig, K.-H. Schleifer (2009) Fluorescence in situ hybridization for intracellular localization of nifH mRNA Syst. Appl. Microbiol., 32 (2009), pp. 186-192

J.R. Moffitt, S. Pandey, A.N. Boettiger, S. Wang, X. Zhuang (2016) Spatial organization shapes the turnover of a bacterial transcriptome eLife, 5 (2016), p. e13065

H. Kawamoto, T. Morita, A. Shimizu, T. Inada, H. Aiba (2005) Implication of membrane localization of target mRNA in the action of a small RNA: mechanism of post-transcriptional regulation of glucose transporter in Escherichia coli. Genes Dev., 19 (2005), pp. 328-338

J. Fei, D. Singh, Q. Zhang, S. Park, D. Balasubramanian, I. Golding, C.K. Vanderpool, T. Ha (2015) RNA biochemistry. Determination of in vivo target search kinetics of regulatory noncoding RNA. Science, 347 (2015), pp. 1371-1374

G. Dugar, S.L. Svensson, T. Bischler, S. Wäldchen, R. Reinhardt, M. Sauer, C.M. Sharma (2016) The CsrA-FliW network controls polar localization of the dual-function flagellin mRNA in Campylobacter jejuni. Nat. Commun., 7 (2016), p. 11667

V.T. dos Santos, A.W. Bisson-Filho, F.J. Gueiros-Filho (2012) DivIVA-mediated polar localization of ComN, a posttranscriptional regulator of Bacillus subtilis J. Bacteriol., 194 (2012), pp. 3661-3669

Answer

We have added the following sentence to the MS section describing rRNA contacts with protein-coding genes:

“The relatively weak correlations observed in our data may reflect the fact that certain mRNAs migrate for localized translation to specific cellular locations (such as the membrane or poles) where the proteins encoded by these mRNAs are required, rather than staying close to transcription sites[23-25]”.

The references cited are as follows:

23 Nevo-Dinur, K., Nussbaum-Shochat, A., Ben-Yehuda, S. & Amster-Choder, O. Translation-independent localization of mRNA in E. coli. *Science* **331**, 1081-1084, doi:10.1126/science.1195691 (2011).

24 Dugar, G. *et al.* The CsrA-FliW network controls polar localization of the dual-function flagellin mRNA in Campylobacter jejuni. *Nature communications* **7**, 11667, doi:10.1038/ncomms11667 (2016).

25 Moffitt, J. R., Pandey, S., Boettiger, A. N., Wang, S. & Zhuang, X. Spatial organization shapes the turnover of a bacterial transcriptome. *eLife* **5**, doi:10.7554/eLife.13065 (2016).

Comment

#2.- With regards to the following statement (lines 125-128):

“The absence of enrichment at the parental gene may reflect the high stability of tRNA and rRNA molecules and their rapid release from the parental gene upon transcription termination and diffusion to the place of functioning. For example, rRNA might be rapidly assembled into ribosomal particles and relocate…”

It is difficult to judge this since, due to the filtering of non-uniquely mapped DNA, we have limited data related to rRNAs mapping to their own loci. Was there an independent evaluation made in that regard?

Answer

In this revised version of the MS, we included additional analysis of preferences of RNAs for long- and short-range interactions performed without taking into account RNA contacts with rRNA operons (now Supplementary Fig. 7). In this variant of analysis, rRNAs demonstrated similar or even higher frequencies of contacts in the interval ±5 kb from the encoding gene compared to “5-50” and “50-500” intervals, however the degree of enrichment was much lower than that observed for mRNAs.

To support the notion that the observed depletion of rRNAs from their parental loci in *E. coli* and *B. subtilis* is due to a poor mappability of DNA reads along rRNA operons, we added the following sentence:

"When we did not take into account contacts with rRNA operons and considered contacts only with their flanking regions, the frequency of rRNA contacts in the parental interval became similar to the frequency of rRNA contacts in more remote genomic intervals (Supplementary Fig. 7a, b)."

Also, while working on this revision, we noticed and corrected inaccuracies made in displaying violin plots in the original version of Fig. 1h, i. The changes are little and do not influence interpretation of the results.

Comment (continued)

The figure provided in the rebuttal actually suggests that there are hundreds of thousands of rRNA-contacts with the rRNA operons, which would have earned a presence close to the top list of supplementary data 1,2,3. Thus, it is not clear why the rRNAs are presumed to leave their site of synthesis rapidly.

Answer

We note that the figure provided in the rebuttal letter presented the numbers of rRNA contacts with the whole genome rather than separate operons. In terms of the number of contacts with the parental locus many rRNA also rank high on the contact list, showing hundreds and thousands of contacts with the parental locus. To support the notion that rRNAs do actually show some preference for their parental loci, but not as much as mRNAs, we have rephrased the following sentences:

Previous version: "In contrast to mRNAs, rRNAs and tRNAs demonstrate widespread distribution and are typically not enriched at the place of their synthesis (Fig. 1g-i). The absence of enrichment at the parental gene may reflect…"

New version: "In contrast to mRNAs, rRNAs and tRNAs demonstrate widespread distribution and are not markedly enriched at the place of their synthesis (Fig. 1g-i, Supplementary Fig. 7). The absence of pronounced enrichment at the parental gene may reflect…"

Comment (continued)

Also, to provide further information on the rRNA operon "mappability" challenges, I would suggest to add to supplementary materials the figure (about rRNA operons from E. coli) provided in your rebuttal together with that portion of your text (with an additional introductory sentence about the uniquely mapped sequence filtering):

"So, we do not assign 1/7 or 1/10 of the contacts to each rRNA operon. We work with contacts that were unambiguously assigned to particular rRNAs. The contact numbers for individual RNAs can be found in excel spreadsheet Supplementary Data 1 (E. coli), Supplementary Data 2 (B. subtilis), and Supplementary Data 3 (T. adornatum). Here is an output for rRNAs in E.coli:

Figure S4 (correction for this figure, even if the Y-axis scale is shown in a log-scale, the number of contacts is not indicated in log, so the "lg" unit should be deleted)

It is seen that the number of mapped (unique) contacts for different rRNAs belonging to the same operon and for different operons varies drastically. Inspection of RNA-DNA contacts maps also shows a huge variation in the number of contacts identified for different portions of the same rRNA molecule. For example, Supplementary Figure 3 shows the contacts of 16S rRNA from the rrnB rRNA operon. It can be seen that the contacts are only detected for the end of 16S rRNA. In view of such variations in contact numbers caused by multiple mapping issues, it is difficult to consider the absence or small number of contacts of some rRNAs as an indication of low transcription level of the corresponding operon."

Answer

As suggested by the editor, we added the data on the number of contacts identified for individual rRNAs in the genomes of all microbial species studied (now Supplementary Fig. 2). We corrected the Y-axis scale in this figure by removing the "lg" unit and added minor ticks to indicate a logarithmic spacing. The necessary explanations addressing the "mappability" of the rRNA operon are given in the figure legend which runs as follows:

"**Supplementary Fig. 2. Number of contacts identified for individual rRNAs in experiments with *E. coli* (a), *B. subtilis* (b) and *T. adornatum* (c).** Presented is the total number of rRNA contacts in the genome. We note that our read mapping procedure filters out RNA-DNA ligation products whose RNA portion, or DNA portion, or both, map more than one time on the genome and retains only uniquely mapped RNA-DNA contacts. With this mapping procedure, a large fraction of rRNA contacts remain unidentified in *E. coli* and *B. subtilis*, because these species contain several copies of rRNA operons in their genomes (7 and 10, respectively), and many rRNA fragments present in RNA-DNA ligation products cannot be unambiguously assigned to a

particular rRNA. Moreover, the used mapping procedure filters out not only many rRNA-DNA ligation products representing contacts of rRNA with different genomic regions, but also many RNA-rDNA ligation products representing contacts of different RNAs (including rRNA itself) with rRNA operons. An alternative strategy would be to allow multiple mapping and then to assign 1/7 or 1/10 of the identified rRNA contacts to each rRNA operon. However, we did not use this strategy because we wished to preserve information about the origin of rRNA fragments. The different yield of unique mappings appears to be a primary factors behind a drastic variation in the number of RNA-DNA contacts identified for different rRNAs (**a,b**) and for different portions of the same rRNA molecule in experiments with *E. coli* and *B. subtilis*. Note, for example, that for the rrnB rRNA operon of *E.coli* the contacts are only detected for the end of 16S rRNA (see Supplementary Fig. 4). The different expression level of rRNA operons may also contribute to the observed variation in the contact number determined for different rRNAs."

In this revised version of the MS, we also indicated the branch of the RedClib pipeline that was used for read processing. In the section "Data availability" of the MS and in the section "Data" of the Reporting Summary we replaced "The code for read processing is available as RedClib on github: https://github.com/agalitsyna/RedClib" with "The code for read processing is available as RedClib on github: https://github.com/agalitsyna/RedClib/tree/redc-bridge-variants".
